# Long-acting management of diabetes and associated complications using an injectable thermosensitive hydrogel incorporating IgG-conjugated GLP-1RA

**DOI:** 10.7150/thno.120844

**Published:** 2026-01-01

**Authors:** Hancheng Wang, Zhiyong Chen, Siyi Gu, Yaoben Wang, Yang Wang, Caiyun Gao, Jiayue Shi, Jiandong Ding, Qinghua Wang, Lin Yu

**Affiliations:** 1 State Key Laboratory of Molecular Engineering of Polymers, Department of Macromolecular Science, Fudan University, Shanghai 200438, China.; 2 Department of Endocrinology and Metabolism, Huashan Hospital, Shanghai Medical School, Fudan University, Shanghai 200040, China.; 3 Innogen Pharmaceutical Technology Co., Ltd., Shanghai 201203, China.

**Keywords:** type 2 diabetes mellitus, glucagon-like peptide-1 receptor agonists, injectable hydrogel, long-acting formulation, glycemic control

## Abstract

**Rationale**: The emergence of glucagon-like peptide-1 receptor agonists (GLP-1RAs) has advanced diabetes management. Nevertheless, frequent administration remains a challenge, even with weekly formulations. Herein, we developed a sustained-release hydrogel-based delivery system for Efsubaglutide Alfa (Suba), an IgG-conjugated GLP-1RA, designed to alleviate treatment burden and enhance patient adherence.

**Methods:** A series of biodegradable poly(lactic acid-*co*-glycolic acid)-poly(ethylene glycol)-poly(lactic acid-*co*-glycolic acid) (PLGA-PEG-PLGA) triblock copolymers were synthesized, and a thermosensitive PLGA-PEG-PLGA hydrogel with suitable sol-gel transition temperature and *in vivo* degradation profile was selected for the fabrication of the Suba-loaded hydrogel system (Suba@T-gel). The pharmacokinetic and pharmacodynamic profiles following subcutaneous administration of Suba@T-gel were evaluated in multiple rodent models.

**Results:** *In vivo* non-invasive imaging and pharmacokinetic studies showed that a single subcutaneous injection of Suba@T-gel enabled sustained release of Suba for over three weeks. This prolonged release profile is attributed to moderate Suba-polymer interactions and the large molecular size of Suba, which facilitate sustained drug release through a carrier degradation-controlled mechanism. In diabetic murine models, a single administration of Suba@T-gel achieved stable glycemic control for three weeks. Furthermore, the continuous liberation of Suba remarkably enhanced insulin secretion, reduced glycosylated hemoglobin levels, and improved pancreatic function in diabetic mice. Additionally, this system ameliorated diabetes-related complications by improving lipid metabolism, reversing hepatic steatosis and enhancing nerve fiber density.

**Conclusions:** The Suba@T-gel system represents a promising strategy for long-acting management of diabetes and substantial improvement in patient compliance.

## Introduction

Diabetes mellitus, which has emerged as one of the most critical public health challenges of our era, is a chronic endocrine and metabolic disorder characterized by persistent hyperglycemia and a myriad of associated complications, such as cardiovascular disease, neuropathy and nephropathy 1-3. According to the International Diabetes Federation (IDF), more than 500 million people worldwide are currently afflicted with diabetes, with type 2 diabetes mellitus (T2DM) accounting for over 90% of cases 1, 2, 4. Most patients with diabetes will develop complications, and cardiovascular complications are the leading cause of diabetes-related mortality 2, 4. Therefore, achieving adequate glycemic control while effectively managing diabetes complications is the key to extending patients' lives and improving their quality of life. Unfortunately, nearly half of individuals with T2DM fail to achieve adequate glycemic control, with poor medication adherence—largely due to the burden of frequent invasive injections—being a major contributing factor 5.

Glucagon-like peptide-1 (GLP-1) is an incretin hormone secreted by intestinal L-cells that enhances insulin secretion in a glucose-dependent manner while suppressing glucagon release 6. Meanwhile, GLP-1 can retard gastric emptying and promote satiety, thereby contributing to weight loss—one of the critical factors in managing T2DM 7, 8. Additionally, numerous studies have disclosed that GLP-1 expression may bring benefits to multiple organs beyond glucose regulation 7, 9-14. However, the very short half-life (*t*_1/2_) of endogenous GLP-1, due to its rapid degradation by dipeptidyl peptidase-4 (DPP-4), limits its therapeutic utility 15. To surmount this limitation, various GLP-1 receptor agonists (GLP-1RAs) have been exploited through structural modifications to extend their *t*_1/2_ and thus enhance their duration of action and efficiency *in vivo* 16-19.

Currently, GLP-1RAs have replaced insulin as the preferred injected antidiabetic drugs for the management of T2DM, as recommended by major international clinical guidelines 20. Different from earlier generations of GLP-1RAs, such as exenatide, liraglutide and lixisenatide, which require administration once or twice daily 21-23, long-acting GLP-1RAs offer superior glycemic control for T2DM management while reducing the burden of frequent injections 17, 24. Efsubaglutide Alfa (Suba), developed by Innogen Pharmaceutical Technology Co., Ltd., is a novel long-acting GLP-1RA designed to extend *in vivo t*_1/2_ and dosing intervals while maintaining efficacy. It is a recombinant GLP-1 analog conjugated to the Fc domain of IgG2, which enhances its stability and reduces renal clearance 25, 26. Preclinical studies have demonstrated its ability to improve glucose homeostasis, pancreatic function, and metabolic health in diabetic rodent and non-human primate models 26, 27. In clinical trials, Suba has shown robust reductions in glycated hemoglobin (HbA_1c_), durable glycemic control, and a prolonged *t*_1/2_ compared to existing GLP-1RAs 17, 24, 27, 28. In January 2025, it received regulatory approval in China as a once-weekly formulation for blood sugar control in adults with T2DM.

While weekly GLP-1RA formulations improve adherence compared to daily injections, studies suggest that the difference in patient compliance is not as significant as expected 5, 29. This highlights the urgent need to develop ultra-long-acting formulations that can further reduce injection frequency, such as monthly administration. Unlike insulin, GLP-1RAs are ideal candidates for sustained-release delivery systems due to their glucose-dependent mechanism, which minimizes hypoglycemia risk 21, 30, 31. Therefore, further prolonging the duration of GLP-1RAs based on sustained-release carriers holds considerable clinical demand and economic benefits.

Over the past decade, injectable thermosensitive hydrogels comprised of poly(ethylene glycol) (PEG)-polyester 32-36, PEG-polypeptide 37-40, poly(phosphazene)s 41, 42 and other amphiphilic polymers 43-45 have garnered growing attention as promising contenders for sustained drug delivery on account of their good biocompatibility and minimally invasive administration. Generally speaking, these systems are injectable sols at low or room temperatures, facilitating the encapsulation of fragile therapeutic agents such as proteins and peptides through simply mixing. This loading process not only prevents the denaturation/degradation of proteins and peptides but also minimizes drug loss 46, 47. Once injected into the body, the therapeutic payloads are spontaneously wrapped within the in-situ forming hydrogels as a consequence of the body temperature-triggered sol-gel transition, followed by continuous release of therapeutic agents from the gel depots via drug diffusion or/and carrier polymer degradation.

**Figure 1:**
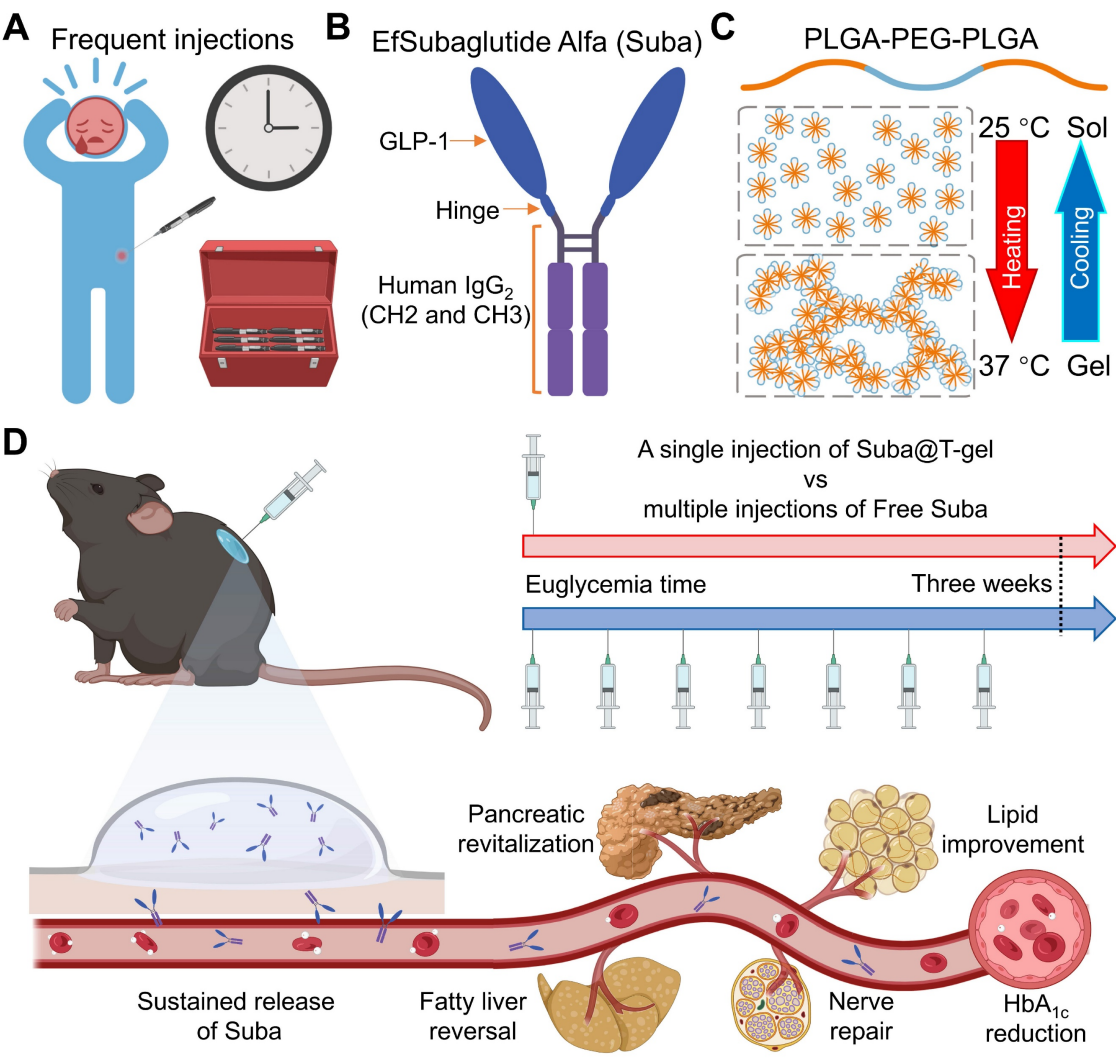
Schematic diagram of the sustained-release formulation of Suba for extended glycemic control and comprehensive management of diabetes-related complications. (A) Conventional frequent injections pose a significant burden on patients and impact adherence. (B) Structural composition of Suba, an IgG-conjugated GLP-1RA. (C) Thermoreversible sol-gel transition feature of PLGA-PEG-PLGA hydrogel. A sol is a suspension of micelles formed by PLGA-PEG-PLGA polymers, while in the gel state, a percolated network is created through micellar aggregation. (D) A single administration of the sustained-release formulation of Suba achieves prolonged glycemic control for three weeks in diabetic mice and effectively ameliorates multiple diabetes-related complications, including dyslipidemia, fatty liver, and neuropathy.

In this study, we developed a hydrogel-based sustained-release formulation of Suba that alleviates treatment burden by substantially reducing the frequency of injections while maintaining efficacy. Given that OncoGel®, a thermosensitive poly(lactic acid-*co*-glycolic acid)-poly(ethylene glycol)-poly(lactic acid-*co*-glycolic acid) (PLGA-PEG-PLGA) polymer hydrogel loaded with paclitaxel was ever approved for Phase IIb clinical trials, this reflects regulatory recognition of the biocompatibility and safety profile of PLGA-PEG-PLGA hydrogels 48, 49. Therefore, we selected this type of hydrogel as the delivery carrier for Suba. By screening a series of PLGA-PEG-PLGA triblock copolymers with varying PEG/PLGA and lactic acid (LA)/glycolic acid (GA) ratios, a thermosensitive PLGA-PEG-PLGA hydrogel suitable for the sustained release of Suba was confirmed. Subsequently, the interactions between Suba and PLGA-PEG-PLGA polymers was investigated, and the sustained release of Suba was detected *in vitro* and* in vivo*. The pharmacokinetic profile and therapeutic efficacy of the sustained-release formulation of Suba were evaluated in four rodent models using multiple observation methods. Finally, its benefits to diseased organs and diabetes-related complications were determined. Overall, this hydrogel-based sustained-release system has the potential to enable once-monthly dosing, offering a clinically viable and patient-friendly alternative for T2DM management. Figure 1 summarizes the study rationale and objectives.

## Results

### Synthesis and characterization of PLGA-PEG-PLGA copolymers

**Figure 2:**
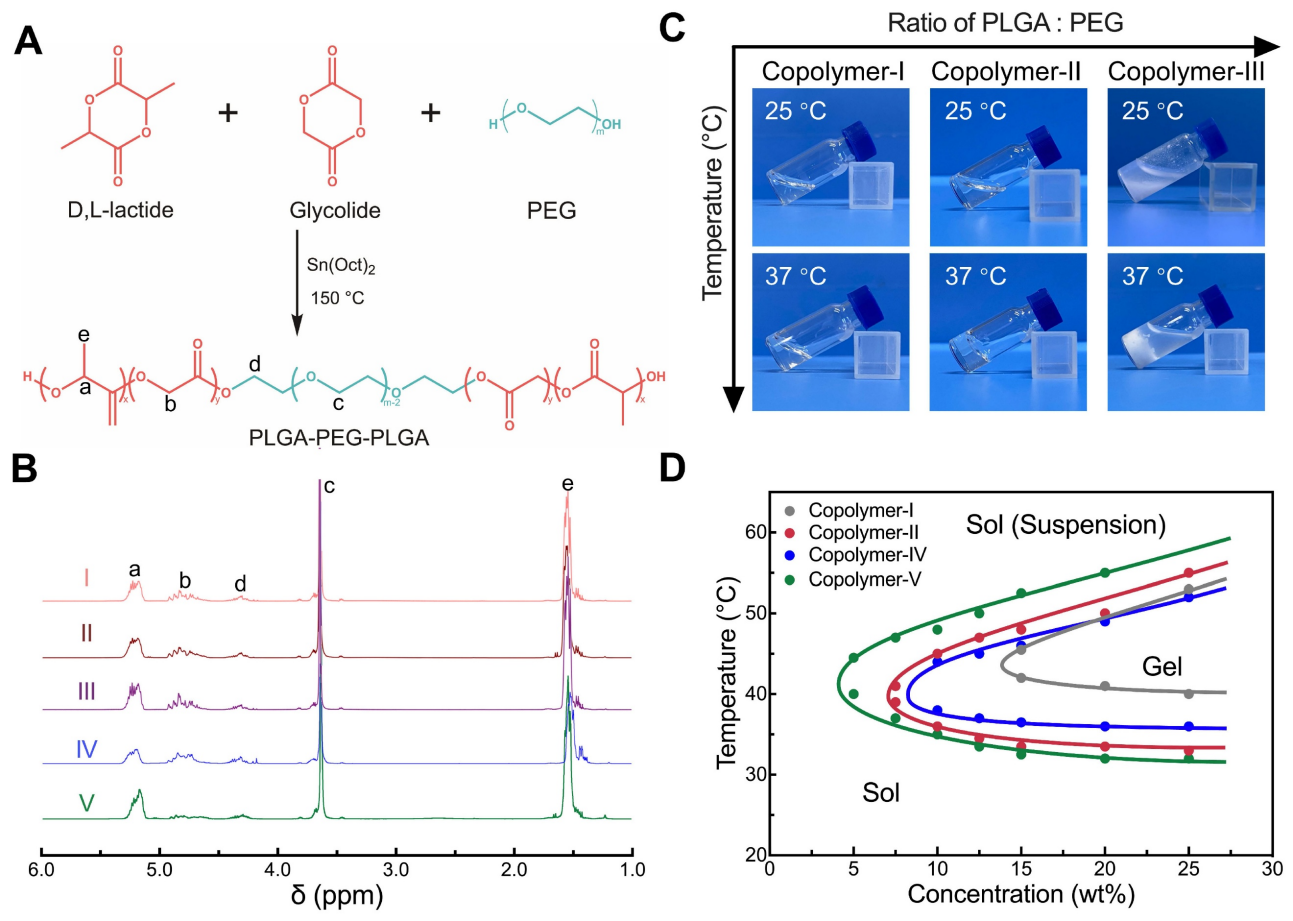
Synthesis of PLGA-PEG-PLGA and characterization of the hydrogels. (A) Synthesis route of PLGA-PEG-PLGA. (B) ^1^H-NMR spectra of PLGA-PEG-PLGA triblock copolymers with different MWs and segment ratios. (C) Optical images of different copolymer/water systems at room temperature or body temperature. (D) Phase diagrams of different copolymer/water systems illustrating the sol-gel transition at varying concentrations and temperatures.

A series of PLGA-PEG-PLGA triblock copolymers, designated as Copolymer I to V, with various molecular weights (MWs) and LA/GA ratios were synthesized via a one-step reaction, as depicted in Figure 2A. The compositions and number-average MWs (*M*_n_s) of the synthesized copolymers were determined using their ^1^H nuclear magnetic resonance (^1^H-NMR) spectra (Figure 2B). Gel permeation chromatography (GPC) analysis further revealed that the synthesized samples exhibited relatively small molar mass dispersity (*Đ*_m_) values, signifying that their purity was sufficient to investigate their physicochemical properties. The basic parameters of the resulting copolymers are summarized in Table 1.

### Temperature-responsive sol-gel transition of aqueous polymer solutions

Among the synthesized copolymers, Copolymer-III, containing the longest PLGA block, was overly hydrophobic and failed to dissolve in water, making it unsuitable for further investigation. The remaining four PLGA-PEG-PLGA copolymers exhibited solubility in water and spontaneously underwent sol-gel transitions with increasing temperature (Figure 2C-D). Among them, Copolymer-I remained in a sol state at body temperature, suggesting its inappropriateness for *in vivo* applications. In contrast, the other three systems (Copolymer-II, IV, and V) formed semi-solid hydrogels at body temperature, indicating their potential as drug delivery carriers. Notably, despite having comparable MWs, their gelation windows and gel strengths were influenced by the LA/GA ratio in the PLGA block. A higher proportion of LA units resulted in a lower sol-gel transition temperature (*T*_gel_), a wider gelation window and an increased gel modulus (Figure 2D and S1).

### Biocompatibility and biodegradation of thermosensitive hydrogels

**Figure 3:**
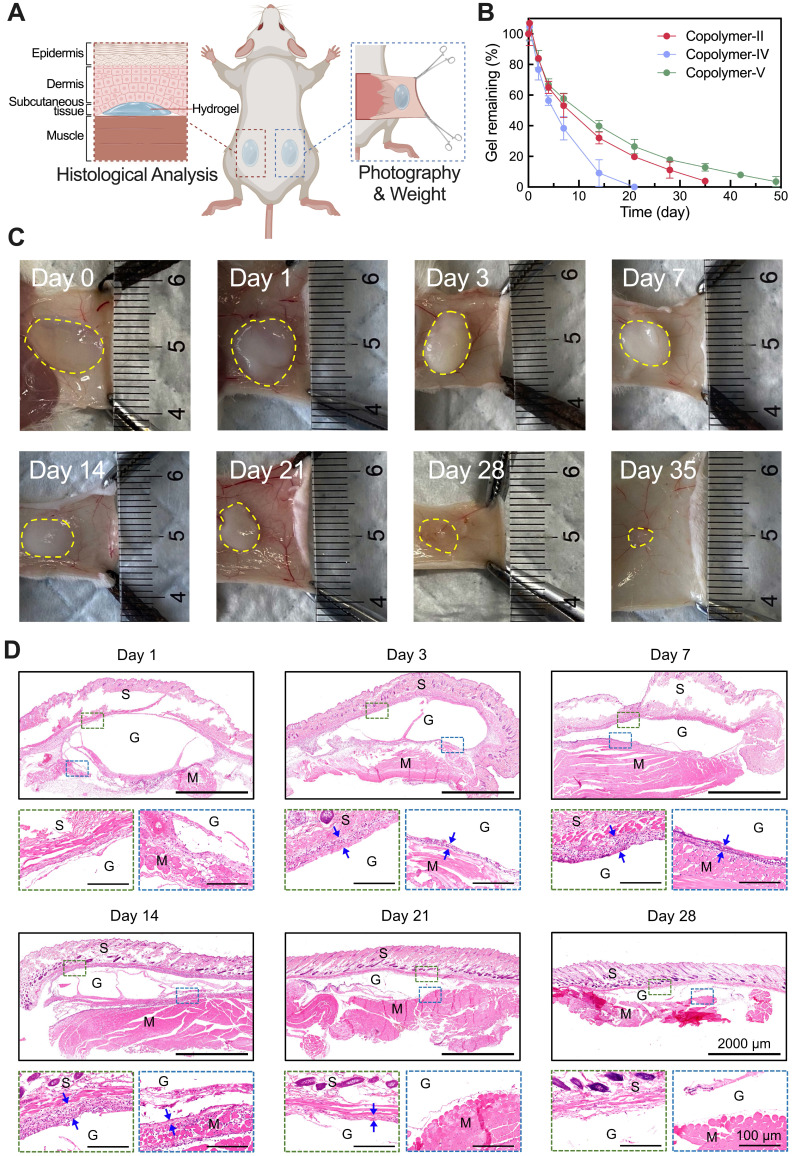
*In vivo* degradation and biocompatibility of PLGA-PEG-PLGA hydrogels. (A) Schematic illustration of subcutaneous injection of hydrogel and subsequent sampling and analysis. (B) Degradation profiles showing changes in the remaining mass of injected hydrogels over time (n = 4). (C) Optical images of residual T-gel at indicated time points. The yellow dashed lines delineate the contour of the residual gel. (D) H&E-stained sections of tissues containing residual T-gel at indicated time points. S: skin, M: muscle, and G: residual gel. The blue arrows denote the generated fibrous layer, which is barely observable on day 28.

We explored the *in vivo* persistence of thermosensitive hydrogels composed of 25 wt% aqueous solutions of Copolymer-II, IV, and V in Institute of Cancer Research (ICR) mice. Figure 3A illustrates the injection sites of the hydrogel and the subsequent assessment methods. It was evident that the *in vivo* maintenance of PLGA-PEG-PLGA hydrogel was also contingent on the LA/GA ratio, and a higher proportion of LA units correlated with a slower degradation *in vivo* (Figure 3B). Given that the thermosensitive hydrogel composed of Copolymer-II, denoted as T-gel in this study, had the most suitable degradation period, approximately one month, this system was chosen as the delivery carrier of Suba for following experiments.

Figure 3C portrays the changes in morphology and size of the residual T-gel as a function of degradation time. The hydrogel initially exhibited a transparent state following subcutaneous injection, and then transformed into an opaque state. This change may be ascribed to the erosion of its hydrophilic components 50. Meanwhile, the hydrogel's volume gradually decreased with the effluxion of time. To further monitor the compositional changes of T-gel during degradation, the gel residues collected at predetermined time points were freeze-dried and subjected to ^1^H NMR analysis (Figure S2A). As summarized in Figure S2B, the hydrophilic EG fraction progressively decreased, whereas the hydrophobic LA fraction steadily increased. The GA fraction showed no significant change, likely due to its relatively low initial content. These findings indicate continuous degradation of PLGA-PEG-PLGA polymers and faster absorption of hydrophilic degradation products compared to their hydrophobic counterparts. In addition, our previous study has demonstrated that the degradation products of PLGA-PEG-PLGA polymers are primarily eliminated through the liver, gallbladder, and spleen 50.

Gross anatomical examination of the injection region revealed no signs of edema, necrosis, or suppuration. Subsequently, some residual gels containing surrounding tissues were also harvested for histological analysis, and the results showed a mild inflammatory response at the implantation site, which diminished as the hydrogel degraded (Figure 3D). Additionally, cell counting kit-8 (CCK-8) assay demonstrated that even at concentrations as high as 1000 μg/mL, Copolymer-II did not exhibit significant cytotoxicity against fibroblasts, adipocytes, or islet cells (Figure S3). These findings, combined with its low hemolysis rate (Figure S4) and normal blood routine index of mice after injection (Figure S5), collectively validated its suitability as a drug delivery vehicle.

### Preparation and characterization of the sustained-release Suba formulation (Suba@T-gel)

**Figure 4:**
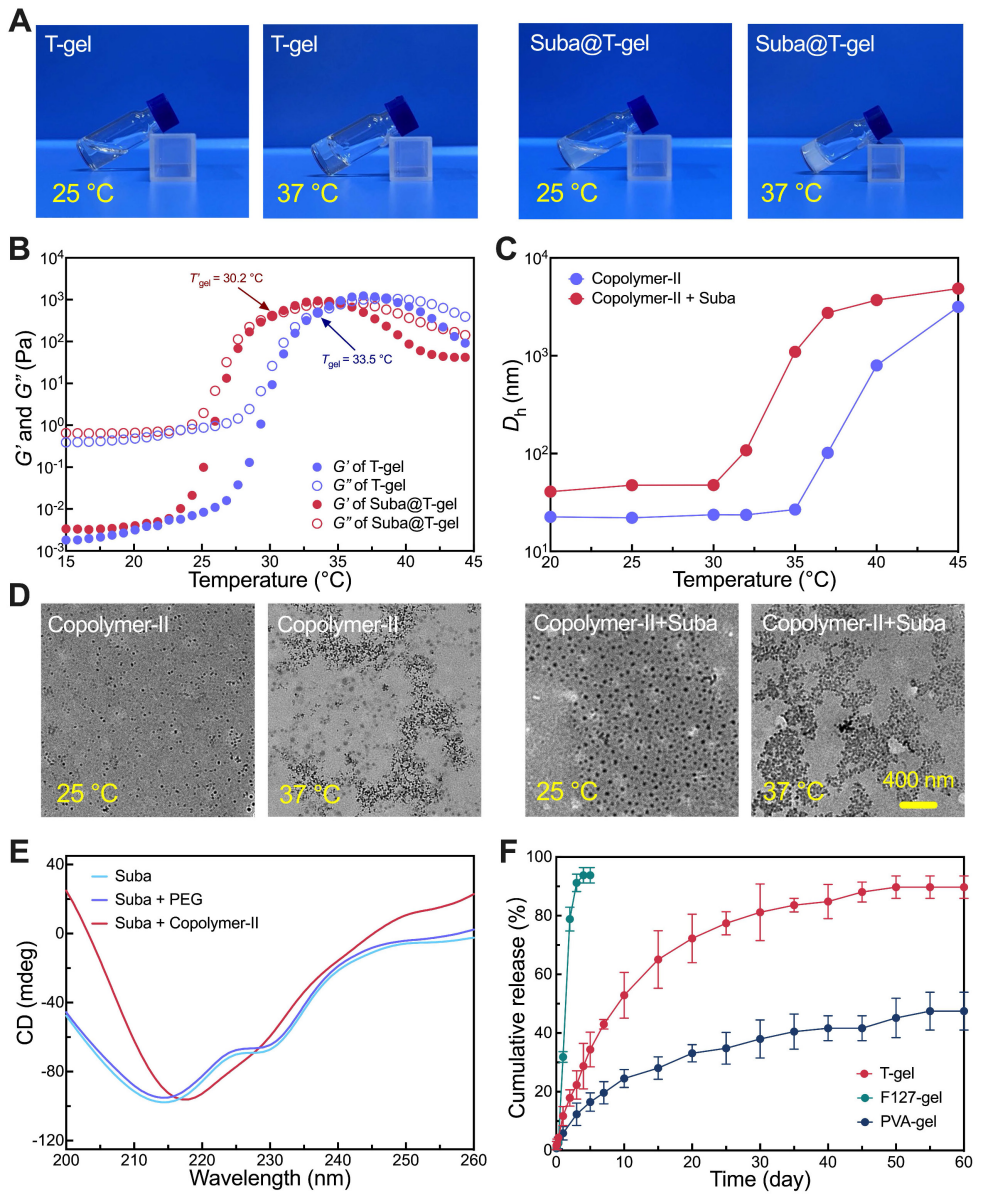
Characterization of Suba@T-gel. (A) Optical images of T-gel and Suba@T-gel at two indicated temperatures. (B) Rheological curves of T-gel and Suba@T-gel as a function of temperature. (C) Hydrodynamic size of Copolymer-II micelles in water with or without Suba at different temperatures. (D) TEM images of micelles formed by Copolymer-II with or without Suba at two indicated temperatures. (E) CD spectra assessing the structural integrity of Suba with and without PEG/Copolymer-II. (F) *In vitro* release profiles of Suba from different hydrogel formulations (n = 3).

T-gel was a low-viscous sol at room temperature, which facilitated the encapsulation of Suba through simple physical mixing, thereby forming the sustained-release formulation Suba@T-gel. As displayed in Figure 4A, Suba@T-gel containing 2.5 mg/mL Suba maintained its sol state at room temperature and converted into a non-flowing gel at physiological temperature; the introduction of Suba reduced the transparency of Suba@T-gel compared with the Suba-free T-gel system. Dynamic rheological measurements further demonstrated that the incorporation of Suba resulted in a reduction in the *T*_gel_ of Suba@T-gel by approximately three degrees relative to the Suba-free T-gel system, but did not obviously affect its modulus (Figure 4B).

The mechanism of temperature-response gelation of PEG-polyester copolymer hydrogels arises from the aggregation of micelles self-assembled by these PEG-polyester copolymers in water, which form a percolated micellar network upon heating 46, 51-54. Dynamic light scattering (DLS) analysis (Figure 4C) and transmission electron microscopy (TEM) observations (Figure 4D) confirmed that the T-gel system follows the same gelation mechanism. We further found that the introduction of Suba did not influence the critical micelle concentration (CMC) of Copolymer-II in water (Figure S6), whereas the micelles incorporating with Suba exhibited an increased size and tended to aggregate at lower temperatures (Figure 4C-D). Obviously, the earlier aggregation of micelles led to a decrease in the *T*_gel_ of Suba@T-gel.

To further explore the possible interactions between the drug and the carrier, circular dichroism (CD) analysis of Suba with or without the carrier polymer was conducted. Pure Suba predominantly exhibited an α-helix structure (Table S1 and Figure 4E). A red shift of CD signal was noticed upon the addition of Copolymer-II, indicating a significant reduction in the α-helix content accompanied by an increase in random coil structures, as shown in Figure 4E. Such structural changes were not observed when Suba was mixed with PEG or replaced by BSA (Figure 4E and S7A). Computational simulation of Suba's amino acid sequence revealed the presence of alternating hydrophilic and hydrophobic regions within Suba (Figure S7B). Consequently, we speculate that amphiphilic Suba participated in the self-assembly process of Copolymer-II in water, leading to an increased micelle size and promoting the formation of the hydrogel network.

The robust stability of Suba was also confirmed. As shown in Figure S8, neither secondary structure changes nor degradation were detected even when a Suba solution was incubated in a water bath shaker at 37 °C for five days. This stability was significantly superior to other GLP-RAs such as exenatide 55.

Subsequently, the *in vitro* release profile of Suba@T-gel was evaluated while employing F127-gel and poly(vinyl alcohol) (PVA)-gel as the controls (Figure S9). The PVA-gel used in this study was fabricated by the classic freeze-thaw method and is well established as a non-degradable hydrogel 56, 57. As illustrated in Figure 4F, Suba experienced a rapid release from F127-gel following the rapid dissolution of the gel matrix in the release medium (Figure S10A). Conversely, due to the high stability of its structural integrity (Figure S10B), Suba exhibited a very slow and incomplete release from PVA-gel, with only 40% of the drug released within 60 days. Unlike the F127-gel and PVA-gel systems, Suba@T-gel displayed a sustained release pattern for up to 60 days without a significant burst release at the first stage or severe incomplete release at the late stage (Figure 4F). Additionally, it should be noted that different from biodegradable T-gel, the non-degradable PVA-gel exhibits considerable risks of polymer accumulation *in vivo* after repeated administration.

### Determination of the optimal drug-loading amount in Suba@T-gel

The oral glucose tolerance test (OGTT) in normal ICR mice is a cost-effective model for evaluating formulation efficacy 58. Therefore, we conducted OGTTs in normal ICR mice to confirm the optimal Suba loading concentration in Suba@T-gel. As illustrated in Figure S11, oral administration of glucose led to a rapid increase in blood glucose levels in the placebo group, whereas the administration of Suba@T-gel at different drug concentrations significantly attenuated postprandial glucose excursions during their respective effective periods. The 2.5 mg/mL formulation maintained glycemic control for up to 22 days, demonstrating a long-lasting hypoglycemic effect (Figure S11). In comparison, the 1.25 mg/mL formulation achieved glycemic control only until day 18, while the 5 mg/mL formulation did not provide an extended duration of control relative to the 2.5 mg/mL dose. Notably, at the 5 mg/mL dose, a few mice exhibited fasting hypoglycemia during the initial phase of administration, suggesting a potential risk of side effects associated with excessive dosing. In contrast, no hypoglycemic symptoms were observed in the 2.5 mg/mL group throughout the study period. These findings support the 2.5 mg/mL formulation as the optimal dosage of Suba@T-gel for sustained glycemic control. Therefore, in all subsequent experiments, the Suba dose in Suba@T-gel was fixed at 2.5 mg/mL.

### Non-invasive monitoring of Suba@T-gel *in vivo*

**Figure 5:**
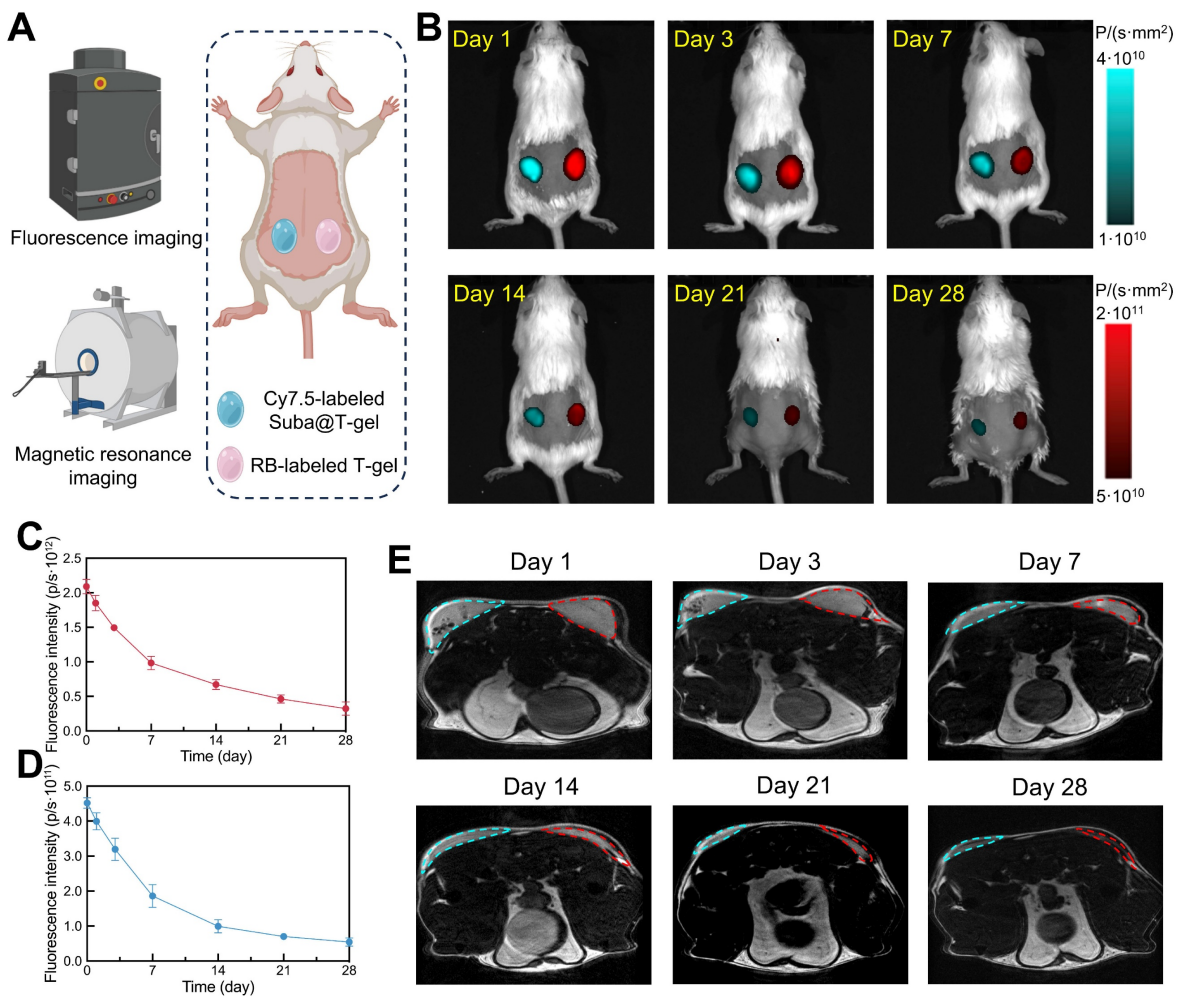
Non-invasive tracing of Suba@T-gel *in vivo.* (A) Schematic illustration of non-invasive monitoring of subcutaneously injected Suba@T-gel and T-gel using fluorescence imaging and MRI. (B) Representative fluorescence images of mice at indicated time points after injection of Suba@T-gel and T-gel. The data was obtained from the same mouse. (C, D) Changes in total fluorescence intensity of RB-labled T-gel (C) and (D) Cy7.5-labled Suba@T-gel as a function of time (n = 3). (E) Representative MRI images of mice at indicated time points after injection of Suba@T-gel and T-gel. The cyan dashed lines indicate the contour of Suba@T-gel, while the red dashed lines represent the outline of T-gel. The data was obtained from the same mouse.

To visualize *in vivo* Suba release, non-invasive fluorescence imaging and magnetic resonance imaging (MRI) were employed. Cy7.5-modified Suba was introduced into Suba@T-gel, while rhodamine B (RB)-capped Copolymer-II as a macromolecular fluorescent probe was incorporated into T-gel. The two systems were injected separately into the left and right sides of the ICR mice's back, as illustrated in Figure 5A. As shown in Figure 5B, the drug or carrier containing fluorescent moieties could be distinctly visualized in the subcutaneous region of the mice. Meanwhile, as the hydrogel degraded or the drug was released, the fluorescence signal intensity gradually diminished. The change trend of total fluorescence intensity for Suba was consistent with that of the carrier polymer (Figure 5C-D), indicating that the degradation of T-gel governed the release of Suba. After approximately three weeks of release, the fluorescence signal of Suba dropped to a very low level. These findings provide an intuitive understanding of *in vivo* Suba release.

MRI is an efficacious approach for observing materials/tissues with high water content, such as hydrogels and cartilage, especially when utilizing *T*_2_-weighted sequences 50, 59. This technique enabled us to clearly visualize the changes in volume and signal intensity of hydrogels over time. As shown in Figure 5E, a gradual reduction in hydrogel volume over time was clearly observed, and the signal intensity decreased significantly about three weeks post-injection, with the hydrogel boundaries becoming indistinguishable after four weeks. Comparisons of transverse plane images between hydrogels with and without Suba showed that the addition of Suba appeared to contribute to water retention within the hydrogel matrix (Figure 5E).

### Pharmacokinetic assessment

**Figure 6:**
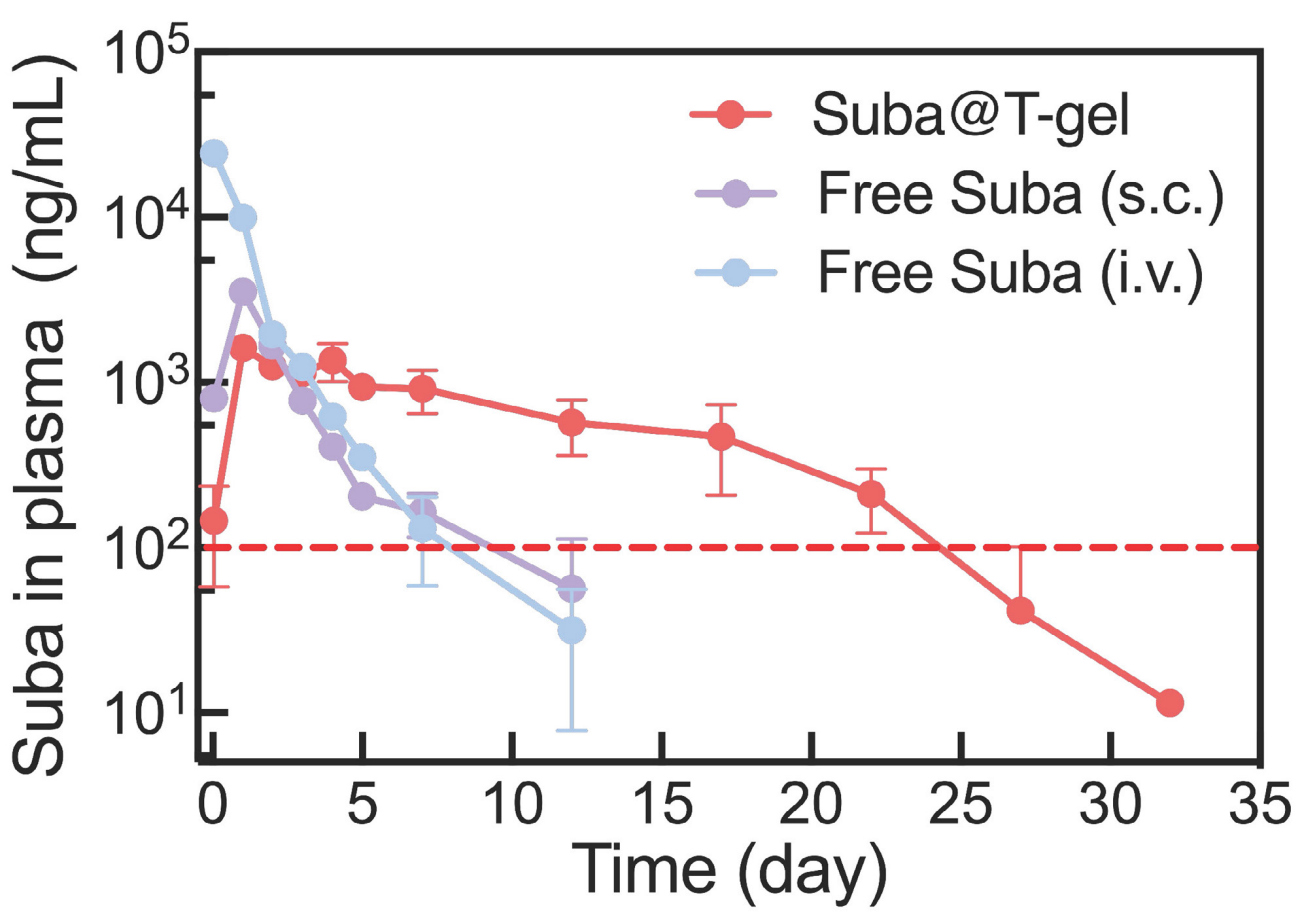
Plasma Suba concentration profiles following a single injection of various Suba formulations in SD rats (n = 3). The same dosage of Suba (2.5 mg/kg) was administrated in the three groups. The red dashed line indicates the minimum effective plasma concentration threshold of Suba. S.c.: subcutaneous injection, and i.v.: intravenous injection.

Pharmacokinetic studies were further performed in Sprague-Dawley (SD) rats to evaluate the maintenance of effective therapeutic concentrations following a single injection of the optimized Suba@T-gel formulation (2.5 mg/mL) (Figure 6). The pharmacokinetic parameters are summarized in Table 2. After a single intravenous administration of Free Suba, plasma drug concentration reached its peak instantaneously and then declined rapidly. In contrast, the time to reach the maximum plasma concentration (*T*_max_) was significantly extended in the subcutaneously administered Free Suba and Suba@T-gel groups. Meanwhile, the maximum plasma concentration (*C*_max_)—a parameter closely associated with the onset of most adverse reactions, particularly gastrointestinal discomfort during the initiation phase 19, was markedly reduced in the Suba@T-gel group, being only approximately half of that observed in the Free Suba (s.c.) group. Furthermore, the *t*_1/2_of Suba in the Suba@T-gel group was significantly longer compared to subcutaneously injected Free Suba. More importantly, a stable plasma concentration of Suba remained above the minimum effective threshold for over 22 days, which was in good agreement with the results of OGTTs (Figure S11).

### *In vivo* long-term hypoglycemic effect

**Figure 7:**
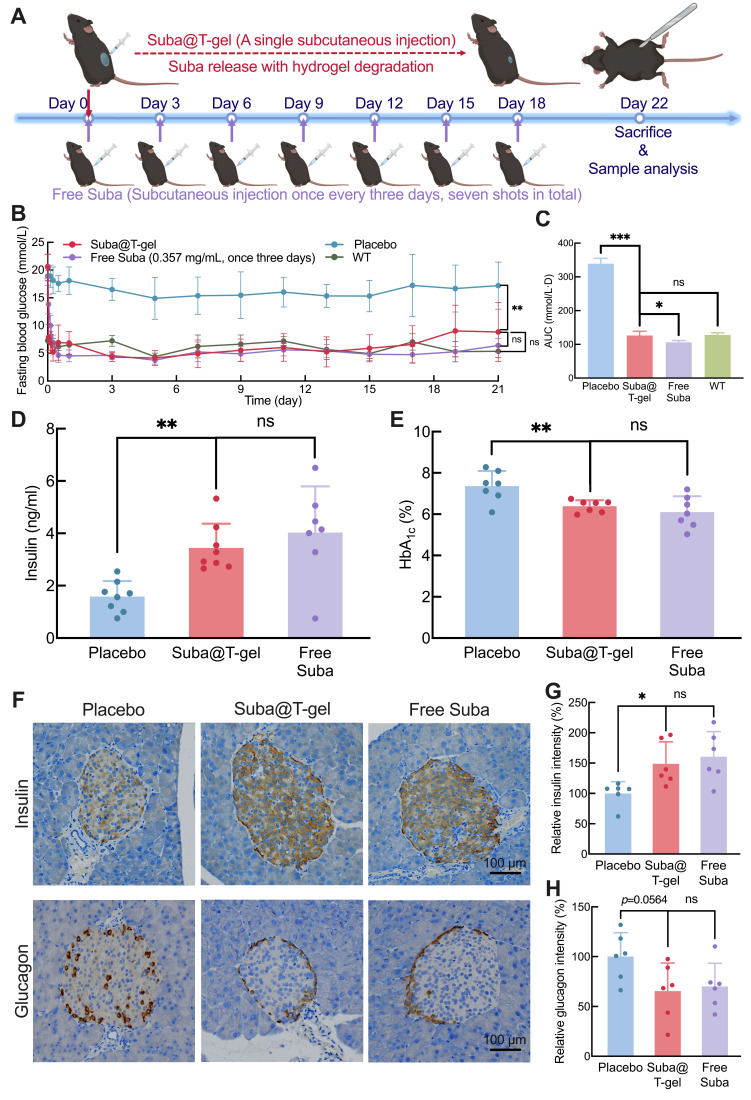
Therapeutic effects of Suba@T-gel in a db/db mouse model. (A) Schematic diagram and timeline of a treatment cycle. (B) Changes in fasting blood glucose levels of mice following different treatments (n = 8). (C) AUC of fasting blood glucose levels after different treatments (n = 8). (D, E) Plasma insulin concentrations and HbA_1c_ levels of mice at the end of various treatments (n = 7-8). (F) Immunohistochemical stained slices of pancreatic tissues at the end of various treatments. (G, H) Quantification of insulin (G) and glucagon (H) immunostaining in pancreatic tissues (n = 6).

We employed a T2DM db/db mouse model to comprehensively investigate the *in vivo* therapeutic effect of Suba@T-gel. As shown in Figure 7A, the mice in the Suba@T-gel group received only a single injection. Notably, a single subcutaneous administration of Free Suba maintained stable glycemic control in db/db mice for only three days (Figure S12). To achieve sustained and stable glucose regulation, Free Suba was administered every three days, consistent with our previous work 26, and the mice in the Free Suba group received a total of seven injections, with the cumulative dosage equivalent to that of a single dose of Suba@T-gel. During the 22-day experiment period, the placebo-treated mice consistently presented high fasting blood glucose levels (Figure 7B). Conversely, the single injection of Suba@T-gel achieved sustained blood sugar control, with fasting blood glucose levels remaining within the normal range until day 21, which was comparable to the effect of seven injections of Free Suba. The statistical analysis of the blood glucose area under the curve (AUC) further supported the above findings (Figure 7C). In addition, these results indicate that biologically active Suba is released from T-gel.

In addition, we carried out OGTTs to evaluate postprandial blood glucose levels in db/db mice. The results showed that, even on day 17, the Suba@T-gel group could significantly inhibit the rise in postprandial blood glucose levels (Figure S13). These findings indicate that Suba@T-gel has the potential to serve as a long-acting formulation for the treatment of diabetes, which not only provides stable control of blood glucose levels but also minimizes the injection frequency.

### Effects of endocrine mediation and islet repair

Given the important increase in random blood sugar levels (> 18.7 mmol/L) three weeks after a single injection of the Suba@T-gel system (Figure S14), all animals were euthanized on Day 22, and blood samples were collected for further analysis. As displayed in Figure 7D, the plasma insulin concentrations in the Suba@T-gel group were significantly higher than those in the placebo group, indicating that the sustained and steady release of Suba effectively promoted insulin secretion. This effect was comparable to that observed in the mice receiving frequent injections of Free Suba. HbA_1c_, formed through an irreversible glycosylation reaction, is recognized as the gold standard for long-term glycemic control and can predict the progression of diabetic symptoms 60. In comparison with the placebo group, both treatments with Suba@T-gel and Free Suba resulted in a significant reduction in HbA_1c_ levels (Figure 7E), with no notable difference between them, suggesting that long-term glycemic control was achieved with a single injection of Suba@T-gel.

Generally, db/db mice exhibit compensatory islet hypertrophy, but in severe cases, β-cell apoptosis occurs, leading to diminished insulin secretion 26, 61, 62. Immunohistochemical analysis of islet tissues revealed that a single injection of Suba@T-gel significantly enhanced insulin secretion while decreasing glucagon release (Figure 7F-H), akin to the Free Suba group. These results imply that the administration of Suba@T-gel contributes to the repair of β cells, thus improving islet function.

**Figure 8:**
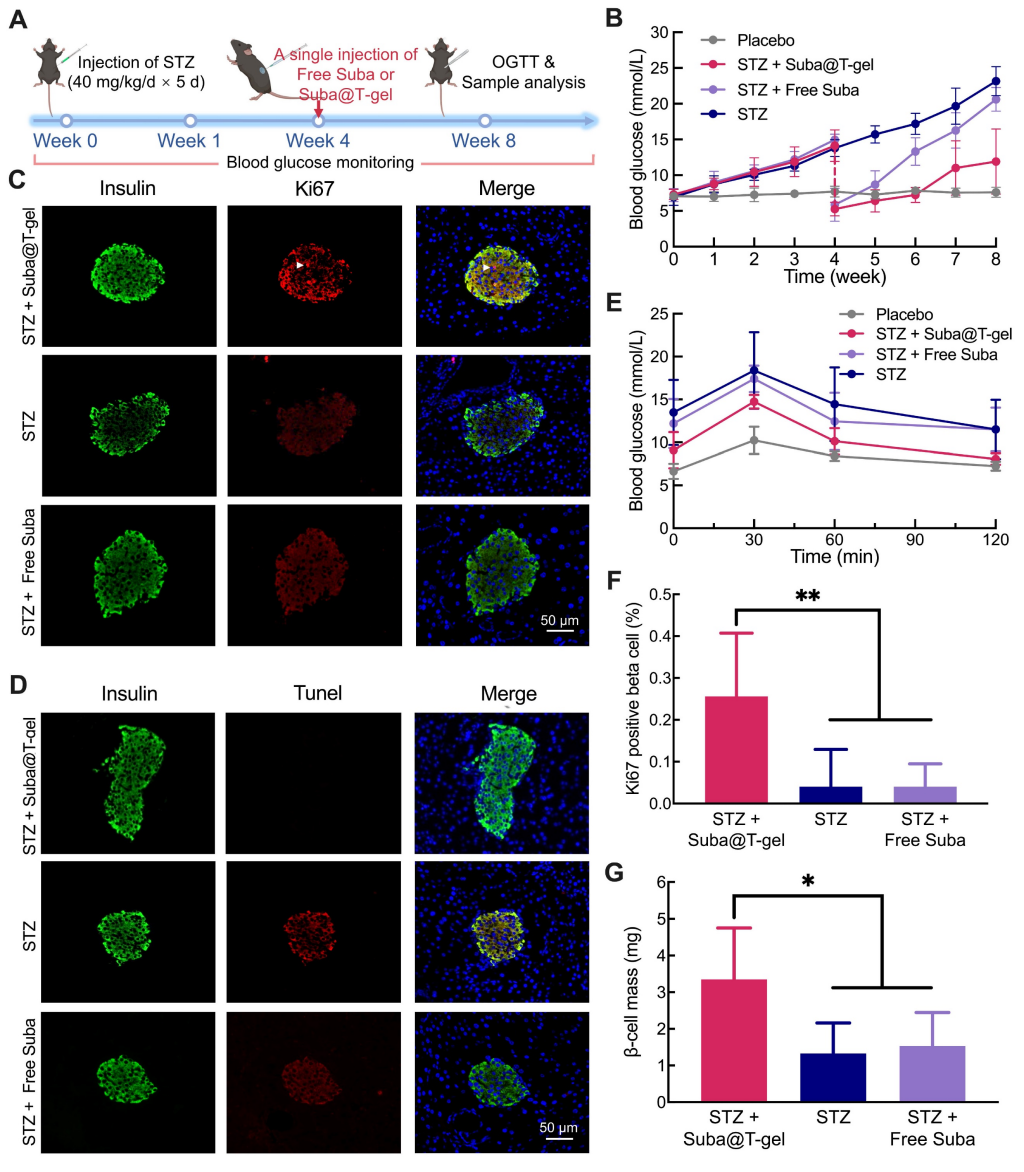
Effects of Suba@T-gel treatment on proliferation of islet cells in STZ-induced diabetic mice. (A) Experimental procedure and timeline. (B) Changes in blood glucose levels after various interventions (n = 8). (C) Insulin (Green) and Ki67 (Red) double stained images of islet cells at the end of various treatments. The white arrows indicate the cell nuclei that are positive for both Ki67 and DAPI staining. (D) Insulin (Green) and Tunel (Red) double stained images of islet cells at the end of various treatments. (E) OGTT results after a single injection of Suba@T-Gel over 4 weeks (n = 8). (F) Quantification of Ki67-positive proliferating cells (n = 6). (G) β-cell mass calculated based on insulin fluorescence staining and islet weight (n = 6).

To further validate whether Suba@T-gel can delay the progression of diabetes resulting from insulin deficiency, we employed another mouse model of islet damage induced by streptozotocin (STZ), which more closely resembles type 1 diabetes. Following five consecutive days of intraperitoneal injection of STZ, the mice exhibited a consistent rise in random blood glucose levels, indicating successful modeling (Figure 8A-B). By the fourth week, treatment with a single injection of Suba@T-gel or Free Suba (2.5 mg/mL) was initiated in a subset of mice. The results presented in Figure 8B showed a significant reduction in blood glucose levels in the STZ + Suba@T-gel group, approaching those of normal mice (the vehicle group). Although blood glucose levels gradually increased over time, they remained significantly lower than those of untreated mice (the STZ group). In contrast, although a single injection of Free Suba led to an initial rapid decrease in blood glucose levels in mice, its efficacy in glycemic control diminished quickly over time. Two weeks after injection, the blood glucose levels in the STZ + Free Suba group were already significantly higher than those in the STZ + Suba@T-gel group. By four weeks, the blood glucose levels in the STZ + Free Suba group had risen to the levels nearly indistinguishable from those in the untreated STZ group.

An OGTT conducted at week 8 further confirmed that both fasting and postprandial glucose levels were lower in the mice treated by Suba@T-gel compared to those in the STZ and STZ + Free Suba groups (Figure 8E).

Subsequently, fluorescence double-label staining of mouse islets revealed that Suba@T-gel treatment promoted β-cell proliferation, as evidenced by the presence of Ki67-positive cells, and enhanced insulin secretion (Figure 8C). Statistical analysis of Ki67 expression and β-cell mass further confirmed that treatment with Suba@T-gel significantly restored islet function (Figure 8F-G), whose effect was markedly superior to that in the STZ + Free Suba group. Additionally, untreated mice and those treated with Free Suba exhibited strong terminal deoxynucleotidyl transferase dUTP nick end labeling (TUNEL) signals, indicative of significant islet apoptosis (Figure 8D). In contrast, TUNEL signals were markedly weaker in the STZ + Suba@T-gel group, confirming that this treatment effectively prevented β-cell apoptosis and preserved islet integrity. These favorable results collectively demonstrate that a single administration of Suba@T-gel, at the same dosage, enables sustained release of Suba, which can more effectively and sustainably prevent chronic pancreatic islet damage compared to a single injection of Free Suba. Taken together, Suba@T-gel not only achieves sustained glycemic control but also promotes the repair and regeneration of islet cells, thereby presenting a promising therapeutic option for diabetes.

### Effects on metabolic health and diabetic complications

**Figure 9:**
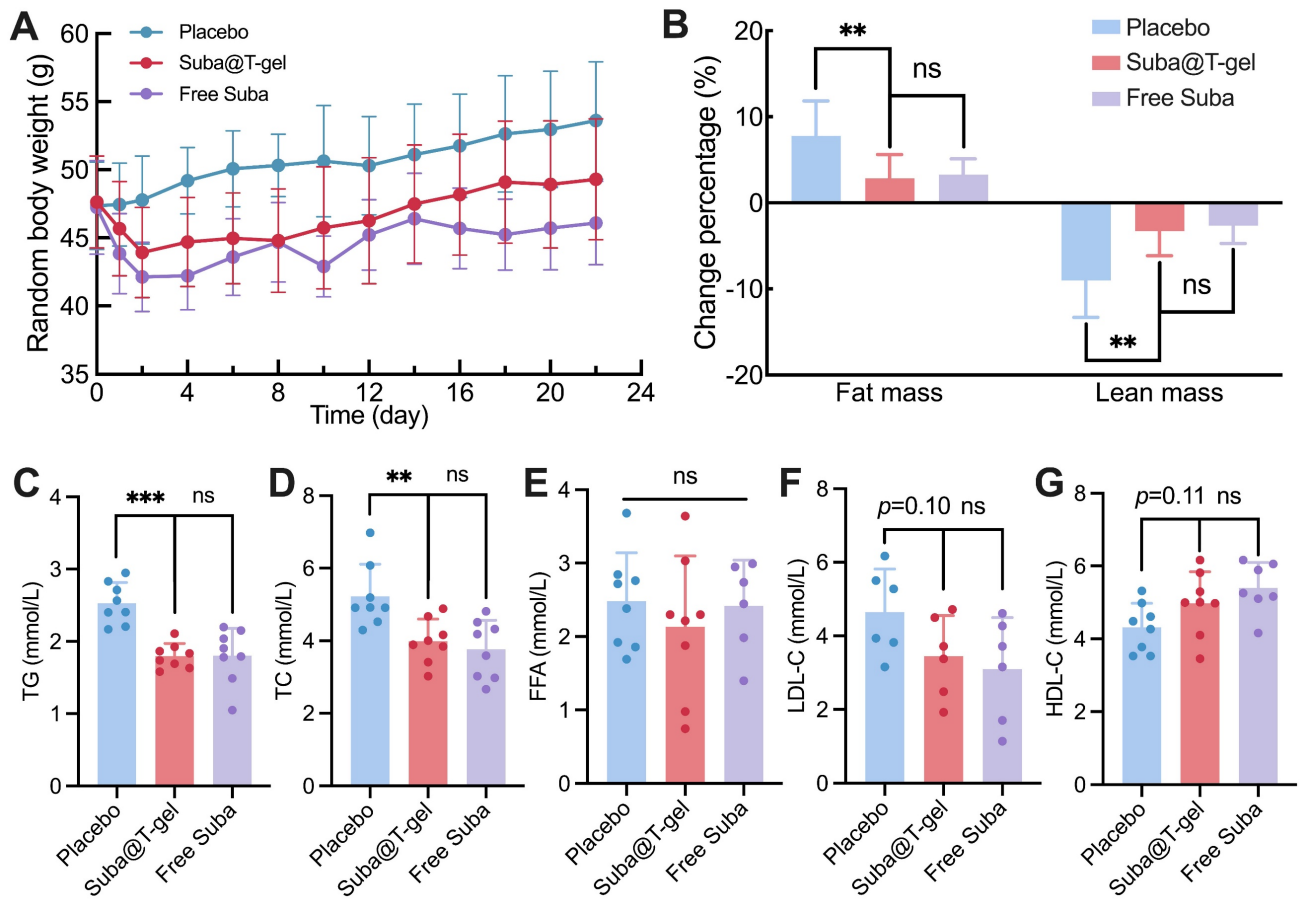
Effects of Suba@T-gel on obesity control and lipid metabolism improvement. (A) Changes in body weight of mice after various treatments (n = 8). (B) Changes in body composition proportions of mice after various treatments (n = 8). (C-G) Serum lipid levels of mice at the end of various treatments: (C) TG, (D) TC, (E) FFA, (F) LDL-C and (G) HDL-C (n = 6-8).

Considering the intricate and close relationship between obesity and diabetes 2, 4, 63, we also monitored the weight changes in db/db mice undergoing various treatments. It was evident that the treatment of Suba@T-gel effectively postponed weight gain in mice compared with the placebo group (Figure 9A), although this effect was slightly inferior to that observed in the Free Suba group. Meanwhile, several abrupt fluctuations in body weight were observed in the Free Suba group, which coincided with the dosing time points. Combined with the pharmacokinetic data showing higher plasma Suba concentrations shortly after the injection of Free Suba (Figure 6), we speculate that the greater body weight reduction is attributable to the higher GLP-1RA exposure, which is consistent with the previous finding 19. Additionally, both Suba@T-gel and Free Suba treatments significantly decelerated the proportion of muscle loss and the proportion of fat gain in mice relative to the placebo group (Figure 9B).

The levels of lipid metabolism markers in blood samples collected on day 22 were detected. Treatment with Suba@T-gel resulted in significant reductions in triglyceride (TG) and total cholesterol (TC) levels, achieving decreases comparable to those seen in the Free Suba group (Figure 9C-D). However, it is noteworthy that serum free fatty acid (FFA) levels remained unchanged regardless of treatment with Suba@T-gel or Free Suba (Figure 9E). High-density lipoprotein cholesterol (HDL-C) is known as a good cholesterol that facilitates the removal of cholesterol from the arterial walls to the liver, whereas low-density lipoprotein cholesterol (LDL-C) is regarded as the “bad” cholesterol because it can cause fatty buildups in the arteries 64, 65. Although no statistically significant difference was noted compared with the placebo group, a slight increase in HDL-C levels and a decrease in LDL-C levels were observed following a single injection of Suba@T-gel (Figure 9F-G).

Metabolic cages were also utilized to visually assess the metabolic alterations in mice (Figure S15A), primarily taking into account that diabetes patients often exhibit abnormal excretion patterns such as polyuria 66. As shown in Figure S15B-C, the administration of Suba@T-gel or Free Suba resulted in a reduction of urine and feces excretion compared with the placebo group. The effect of reduced excretion induced by Suba@T-gel were slightly inferior to that observed in the Free Suba group, which was consistent with the results of weight changes in mice. These findings were further corroborated by AUC calculations (Figure S15B-C).

**Figure 10:**
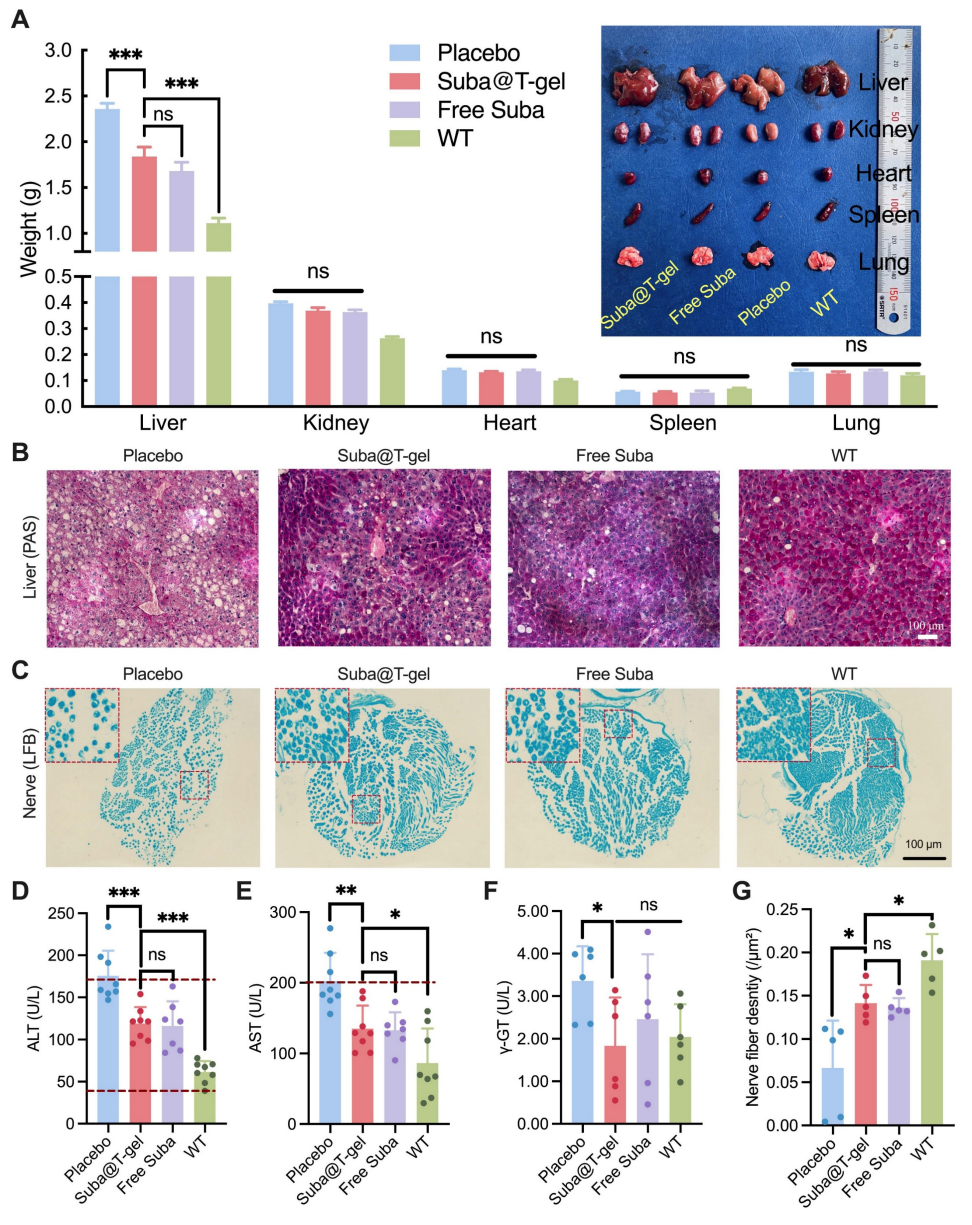
Effects of Suba@T-gel on improvement of diabetic complications. (A) Weight of vital organs of mice at the end of various treatments (n = 8). (B) PAS-stained sections of livers of mice. (C) LFB-stained sections of sciatic nerves of mice. Each dot stained by LFB represents a nerve fiber. (D-F) Serum liver enzyme levels post-treatment: ALT (D), AST (E), and γ-GT (F). The dashed lines indicate normal reference ranges (n = 6-8). (G) Quantification of nerve fiber density in the sciatic nerves (n = 5).

The progression of diabetes often leads to various complications 2, 4, 60. After the db/db mice were sacrificed on day 22, systematical anatomical observations were performed. As shown in Figure 10A, Suba@T-gel treatment markedly reduced liver mass in mice, although they did not completely restore to a normal level. Meanwhile, the livers of untreated mice appeared yellowish-white with a rough, nodular surface, indicative of a fatty liver. In contrast, the livers of mice treated with Suba@T-gel or Free Suba exhibited a healthy brownish-red color with a smooth surface, resembling that of normal mice.

The harvested livers were further stained with periodic acid-Schiff (PAS). As depicted in Figure 10B, a large amount of fat vesicles, characteristic of fatty liver disease, were observed in the untreated db/db mice; however, treatment with Suba@T-gel or Free Suba resulted in a substantial reduction of fat vesicles. Meanwhile, the deeper glycogen-specific staining in the treated mice indicated a marked restoration of hepatic glycogen storage capacity. Additionally, the levels of alanine aminotransferase (ALT), aspartate aminotransferase (AST) and γ-glutamyl transpeptidase (γ-GT) returned to the normal ranges or were comparable to those of wild-type mice (WT) following the administration of Suba@T-gel or Free Suba (Figure 10D-F), indicating the recovery of liver function.

The development of diabetes is also recognized to cause neuropathy, which affects up to 50% of individuals with T2DM 67, and is strongly associated with conditions such as diabetic foot and dementia 68, 69. Consequently, the sciatic nerves of db/db mice were collected and then stained with Luxol Fast Blue (LFB). As shown in Figure 10C, the sciatic nerve fibers in the placebo group were loosely packed. However, after treatment with Suba@T-gel or Free Suba, the nerve fibers regained a compact arrangement. Meanwhile, a higher density of nerve fibers was observed in the Suba@T-gel and Free Suba groups, as presented in Figure 10G. These results suggest that the administration of Suba@T-gel can slow or even reverse diabetic neuropathy.

Last but not least, no significant differences were observed in other major organs among the different treatment groups, including organ mass, gross morphology (Figure 10A), and histological examination (Figure S16). These outcomes suggest that the db/db mouse model used in this study may not be advanced enough to develop complications in these organs, and that treatment with Suba@T-gel did not cause any tissue abnormalities.

## Discussion

Chronic diseases such as diabetes, for which no definitive cure currently exists, represent a significant challenge in modern medicine. The requirement for frequent medication administration often leads to suboptimal patient adherence, further complicating disease management 5, 70. For example, poor medication adherence in T2MD is associated with inadequate blood sugar control, higher medical costs and significantly increased mortality rates 5. Over the last two decades, the emergence of GLP-1RAs has notably improved the quality of living and longevity for individuals with diabetes 7, 9, 29. However, challenges related to medication adherence continue to persist, particularly due to the need for frequent injections 4, 5.

To addressing adherence issues, we exploited a sustained-release system of Suba, spotlighting its potential for long-acting glycemic control. This system achieved effective glycemic control and comprehensive management of diabetes-related complications for over three weeks in various animal models after just a single injection, thereby developing a promising treatment option for diabetes management.

Injectable and thermosensitive hydrogels composed of PLGA-PEG-PLGA copolymers were selected as the carrier for Suba delivery. This choice was based on the consideration that both PEG and PLGA components have approved by the Food and Drug Administration (FDA) for *in vivo* use 71, and the biosafety of PLGA-PEG-PLGA hydrogels has also been confirmed in clinical trials 48, 49. Additionally, the synthesis of PLGA-PEG-PLGA copolymers is straightforward and can be accomplished through a one-step bulk polymerization without the use of organic solvents, and the subsequent purification process is conducted in water, which further enhances their safety and practicability. The PEG/PLGA ratio could determine the* T*_gel_ of the resulting PLGA-PEG-PLGA hydrogel and even influence the formation of a temperature-responsive hydrogel (Figure 2C-D), while the ratio of LA to GA significantly impacted the degradation rate of the hydrogel *in vivo* (Figure 3B). The thermosensitive hydrogel composed of Copolymer-II (T-gel) was employed for the sustained delivery of Suba due to its suitable *T*_gel_ and *in vivo* retention time. The biocompatibility of Copolymer-II and corresponding T-gel were substantiated by *in vitro* and *in vivo* biological experiments (Figure 3 and S3-S5).

It should be noted that previous studies have explored the delivery of GLP-1RAs using PLGA-PEG-PLGA hydrogels, such as exenatide and lixisenatide 55, 60. However, these formulations faced notable challenges. Due to the weak interactions between the drug and the carrier polymer, exenatide exhibited a severe burst release (over 40%) on the first day 55. In contrast, the strong interactions between the drug and the carrier polymer led to a significant incomplete release of lixisenatide (cumulative release amount < 60%) at the late stage 60. In the present study, these challenges were successfully addressed by leveraging Suba's larger molecular size and its moderate co-assembly interactions with PLGA-PEG-PLGA. This ensured a steady, degradation-dependent release profile for up to 60 days *in vitro* (Figure 4F) and for 3 weeks *in vivo* (Figure 6). It should be noted that the differences between the *in vitro* and *in vivo* release profiles are mainly due to the complex physiological environment in the body, which accelerates the *in vivo* degradation of the carrier hydrogel 72. Additionally, T-gel was nearly fully degraded within two weeks following the complete release of Suba *in vivo* (Figure 3B), reducing concerns about polymer accumulation at the injection site and facilitating subsequent injections. These results highlight the advantages of the Suba@T-gel system, extending the duration of GLP-1RA therapy while minimizing undesired fluctuations in drug concentration.

To validate the *in vivo* therapeutic efficacy of Suba@T-gel, we adopted three distinct mouse models: the normal ICR mouse model, the T2DM db/db mouse model and the STZ-induced islet-damaged mouse model, with consistent outcomes observed. Particularly, in the db/db mouse model, the fasting blood glucose levels of mice that received a single injection of Suba@T-gel remained at a normal level until Day 21, while OGTTs demonstrated that the postprandial blood glucose levels were still maintained within a healthy range even towards the end of the single dose of Suba@T-gel (Figure S13). HbA_1c_ is the gold standard for assessing long-term blood sugar control 60. Compared to the placebo group, a single injection of Suba@T-gel plus its subsequently sustained release significantly reduced HbA_1c_ levels, markedly enhanced insulin secretion, and suppressed glucagon release in db/db mice (Figure 7D-H). These positive outcomes were not inferior to those achieved in the Free Suba group but with a drastically reduced injection frequency (7 injections *vs*. 1 injection).

Beyond glycemic control, diabetes is closely associated with β-cell apoptosis, which contributes to disease progression 73. Therefore, alleviating or even reversing β-cell apoptosis is essential for effective diabetes treatment 74. In the STZ-induced islet-damaged mouse model, we found that treatment with Suba@T-gel, compared to a single injection of Free Suba, not only effectively prevented β-cell apoptosis but also remarkably promoted β-cell proliferation. This superior effect of the sustained-release formulation is primarily attributed to its prolonged duration of action and enhanced bioavailability.

Diabetes is closely associated with lipid accumulation and ectopic storage 75, which can cause many lipid-related diseases such as obesity, atherosclerosis and fatty liver disease 2, 75, 76. A single injection of Suba@T-gel treatment effectively mitigated muscle loss and fat accumulation in db/db mice, thereby inhibiting their body weight gain. Despite no changes in FFA levels, significant reductions in TG and TC levels were witnessed following Suba@T-gel treatment, indicating the improvement of overall lipid metabolism (Figure 9C-E). Meanwhile, the treatment of Suba@T-gel elevated HDL-C levels while lowering LDL-C levels, although no significant differences were detected when compared with the placebo group (Figure 9F-G). We conjecture that these benefits would be further amplified with multiple injections of Suba@T-gel. Furthermore, we found that obese db/db mice had developed fatty liver disease. Intriguingly, treatment with Suba@T-gel markedly reduced the weight of liver, improved the liver function and restore the hepatic glycogen storage capacity in db/db mice. Additionally, the sustained release of Suba provided protective effects on the sciatic nerves of db/db mice, suggesting a potential role in delaying or reversing neuropathic complications. These favorable results highlight that, in addition to long-acting blood sugar control, Suba@T-gel holds the potential to delay or even reverse the development of various diabetes-related complications.

Additionally, rodents such as rats and mice have considerably faster metabolic rates in comparison to humans. Consequently, drugs administered to rats or mice often have significantly shorter *t*_1/2_ than those achieved in humans 29, 77. For instance, the *t*_1/2_ of Suba solution in rats is approximately 24 h, while in humans it can be extended to over a hundred hours 18, 27. The pharmacokinetic results demonstrated that a single administration of Suba@T-gel could maintain effective plasma Suba concentrations for over 22 days (Figure 6). Based on prior research regarding the extrapolation of subcutaneous hydrogel depot release durations across different species 29, we speculate that a single injection of Suba@Tgel is likely to provide effective glycemic control for far more than three weeks in human patients.

Nevertheless, this study has certain limitations and further investigation is required. One major limitation is that the system has not yet been tested in larger animal models. The study exclusively utilized rat and mouse models. While these models are widely accepted in diabetes research, their metabolic rates and pathophysiology differ significantly from those of humans. Meanwhile, extrapolating the three-week efficacy in mice to a “monthly” human regimen relies on assumptions based on metabolic scaling. Consequently, pharmacokinetic studies in higher species are critical for evaluating the translational potential and scalability of this therapy system.

## Conclusion

In this study, we successfully developed an injectable hydrogel-based Suba delivery system that significantly reduced injection frequency without compromising efficacy. The moderate co-assembly interactions between Suba and the carrier polymers plus the large molecular size of Suba ensured the continuous and slow release of Suba. In a T2DM mouse model, this system achieved sustained glycemic control for three weeks while effectively managing diabetes-related complications. Given its robust efficacy in preclinical models, this system holds promise for treating T2DM and significant improving patient compliance.

## Materials and Methods

### Animals

Male animals were selected to eliminate the potential interference of sex hormones with the results. For OGTTs, 7-week-old ICR mice were used, while 10-week-old ICR mice were employed for the gel degradation experiment. C57BL/6J mice and db/db mice were 8 weeks old at the start of the experiments. SD rats weighing approximately 500 g were used for pharmacokinetic studies. All rodents had free access to food and water when not undergoing testing and were raised in an environment with a 12-hour day/night cycle. The feeding, anaesthesia, surgical procedures, and handling of experimental animals adhered to the guidelines established by the Experimental Animal Ethics Committee of Fudan University (202101006S).

### Materials

PEG1500, tin(II) 2-ethylhexanoate (Sn(Oct)₂) and Pluronic F127 were purchased from Sigma-Aldrich® (St. Louis, MO, USA). Glycolide and D,L-lactide were obtained from Hangzhou Medzone Biotech Ltd. (Hangzhou, China). PVA was acquired from Aladin® (Shanghai, China). Suba was kindly provided by Innogen Pharmaceutical Technology Co., Ltd. (Shanghai, China). Other chemicals and solvents were purchased from Sinopharm Chemical Reagent Co., Ltd. (Shanghai, China) and used as received without further purification.

CCK-8 kit was purchased from Beyotime Biotechnology (Cat# C0048). Ultra-sensitive mouse insulin ELISA kit and mouse GLP-1 ELISA kit were acquired from Crystalchem (Elk Grove Village, IL, USA; Cat# 90080 and 81508, respectively). TG, TC, HDL-C, LDL-C, and HbA_1c_ assay kits were obtained from Nanjing Jiancheng Bioengineering Institute.

### Synthesis of PLGA-PEG-PLGA copolymers

PLGA-PEG-PLGA triblock copolymers with varying MWs and LA/GA ratios were synthesized by the ring-opening copolymerization of glycolide and D,L-lactide. Sn(Oct)_2_ was selected as the catalyst, while PEG1500 served as the macroinitiator. In brief, a certain amount of PEG was transferred into a three-necked flask and dehydrated under vacuum at approximately 120 °C for no less than 1 h. Subsequently, after cooling the flask to 80 °C, the appropriate amounts of glycolide, D,L-lactide and Sn(Oct)_2_ were introduced into the reaction system. Following purging the flask with argon, the reaction was conducted at 150 °C for 12 h. Thereafter, the crude polymers were washed with 80 °C deionized water at least three times to remove residual monomers and low MW byproducts. Upon lyophilization, the final product was gathered and stored for subsequent use.

### Characterization of copolymers

A 400 MHz ^1^H-NMR spectrometer (Bruker, AVANCE III HD) was used to confirm the chemical structures and MWs of the resulting copolymers. CDCl_3_ was used as the solvent and measurements were conducted at 25 °C. A GPC system (Agilent, 1260) was employed to determine the MWs and *Ð*_M_ values of the samples.

### Preparation of aqueous copolymer solutions

A predetermined amount of copolymer was weighed and transferred into a glass sample vial, followed by the addition of phosphate-buffered saline (PBS). The resulting mixture was stirred using a magnetic stirrer for 4 days. The pH was then measured and adjusted to 7.4.

### Phase diagram

A series of aqueous copolymer solutions with different concentrations were prepared and subsequently transferred into 2-mL tubules for incubation in a water bath. The temperature of the water bath was incrementally increased from 25 °C to 60 °C, with increments of 0.5 °C at each step. After a 10-min equilibrium at each temperature, the tubules containing the samples were inverted in the water bath. No visible flow was observed within 30 s, which was interpreted as a gel. Experiments were repeated three times per sample.

### *In vivo* hydrogel degradation

ICR mice were anesthetized with isoflurane gas, and then 0.2 mL of 25 wt% aqueous solutions of Copolymer-II, IV and V were injected subcutaneously into both sides of the mice's backs. At predetermined time points, some mice were sacrificed for anatomical observation. Subsequently, the optical images of residual hydrogels were captured, and the mass of these residual hydrogels was measured. After lyophilization of these residual hydrogels, the corresponding^ 1^H NMR spectra were obtained. Additionally, tissue samples containing the remaining hydrogels were harvested for histological analysis.

### Rheological study

The temperature-dependent rheological behaviors of aqueous polymer solutions with or without drugs were investigated using a rotational rheometer (Malvern, Kinexus Pro) equipped with a cone plate (cone angle: 1°, diameter: 60 mm, gap: 0.03 mm). Temperature sweep measurements were conducted at a heating rate of 0.5 °C/min over the range of 15 to 45 °C with an oscillation frequency of 10 rad/s.

### TEM observation

A 1 wt% aqueous solution of Copolymer-II with or without Suba was dropped onto the copper meshes. The copper meshes were then placed at room temperature or in a 37 °C air bath overnight. After that, the copper webs were scanned via TEM (FEI, Tecnai G2 20 TWIN).

### CD measurement

A CD spectrometer (Bruker, BIO-logic MOS-450) was used to analyze the secondary structure of samples. CD measurements were conducted at 25 °C over a wavelength range of 200 to 260 nm or 200 to 350 nm. Each spectrum was corrected by subtracting the corresponding ultra-pure water signal as the baseline and the gas signal in the cavity as the background**.**

### Preparation of Suba-loaded hydrogels

For Suba@T-gel, lyophilized Suba powder was added to a 25 wt% aqueous solution of Copolymer-II at an indicated concentration, and the mixture was then stirred magnetically for 24 h until a homogeneous state was achieved.

For Suba@F127-gel, Pluronic F127 was dissolved in PBS to obtain a 20 wt% solution, after which lyophilized Suba powder was incorporated at a concentration of 2.5 mg/mL and then stirred magnetically for 24 h until uniform.

For Suba@PVA-gel, PVA was dissolved in PBS to prepare a 10 wt% solution and Suba was then added at a concentration of 2.5 mg/mL. Next, the system underwent a freeze-thaw cycle consisting of storage at -20 °C for 6 h and subsequent thawing at room temperature for another 6 h.

### *In vitro* drug release

1 mL of Suba@T-gel, Suba@PVA-gel or Suba@F127-gel was introduced into 10-mL release tubes, respectively. The concentration of Suba in each gel was 2.5 mg/mL. Then, the tubes were placed in a water bath shaker (50 rpm) at 37 °C for an equilibration of 10 min. Next, 8 mL of pre-heated PBS solution containing 0.025% NaN_3_ was added as the release medium. At each sampling time point, optical images of the hydrogels were taken and 5 ml of the release medium was withdrawn and replaced with an equal volume of fresh PBS. All collected samples were stored at -20 °C until analysis. After completion of the release experiment, the concentration of Suba in the collected media was quantified using Mouse GLP-1 ELISA kit.

### Fluorescence imaging in mice

RB-modified Copolymer-II was co-dissolved with Copolymer-II in water at a ratio of 1:10,000 to obtain a 25 wt% aqueous polymer solution (RB-gel). Meanwhile, 2.5 μg of Cy7.5-Suba were added to Suba@T-gel, resulting in the formation of Cy7.5-Suba@T-gel. Subsequently, 0.2 mL of RB-gel was injected subcutaneously into the right dorsal region of mice, while the same volume of Cy7.5-Suba@T-gel was injected into the left region. Changes in fluorescence intensity at the injection site over time were tracked using a multi-angle small animal optical *in vivo* imaging system (Biolight Biotechnology Co., Ltd., Aniview Pro). Fluorescence imaging for RB-gel was performed at an excitation wavelength of 560 nm and an emission wavelength of 600 nm, while fluorescence imaging for Cy7.5-Suba utilized an excitation wavelength of 790 nm and an emission wavelength of 810 nm. Each imaging exposure lasted for 1.5 s. The three sets of images were then merged and processed using different pseudocolors.

### MRI in mice

T-gel and Suba@T-gel (0.2 mL each) were subcutaneously injected into the right and left dorsal regions of ICR mice, respectively. At designated sampling time points, the residual hydrogels were imaged using a 7.0T high-field small animal MRI system (SHCG, NOVILA 7.0T). *T*_2_-weighted spin-echo sequences were employed for imaging, with the mouse bladder serving as the reference for localization while capturing both transverse and sagittal views.

### OGTT model

Before the measurements, the mice were fasted for 6 h with free access to water. Following the administration of a glucose solution in mice via gavage at a dose of 1 g/kg, blood samples were collected using the tail clipping method at 0, 30, 60, and 120 min post-administration. Subsequently, blood glucose levels were determined by a standard glucose meter.

### Pharmacokinetic study

SD rats were randomly assigned into three groups (n = 3 per group): intravenous injection of Suba solution (Free Suba (i.v.)), subcutaneous administration of Suba solution (Free Suba (s.c.)), and subcutaneous injection of Suba@T-gel (Suba@T-gel). The dosage of Suba was set at 2.5 mg/mL, with each rat receiving a single injection (0.5 mL). At specific time points, approximately 0.5 mL of whole blood was collected via the tail vein into anticoagulant tubes. Blood samples were centrifuged at 1000 × g for 10 min at 4 °C, after which the upper plasma layer was collected and stored at -80 °C until analysis. Finally, the concentration of Suba in the plasma was quantified using a Mouse GLP-1 ELISA kit.

### Diabetic db/db mouse model

Diabetic db/db mice aged 8 weeks were randomly divided into three groups (eight per group): Placebo (subcutaneous injection of 0.2 mL normal saline every three days), Free Suba (subcutaneous injection of 0.2 mL Suba solution at a concentration of 0.357 mg/mL every three days) and Suba@T-gel (a single subcutaneous injection of 0.2 mL Suba@T-gel (2.5 mg/mL)). The mice in the Placebo and Free Suba groups received a total of seven injections. Meanwhile, another group consisting of eight normal C57 mice with the same age was designated as WT and did not receive any administration. During the 22-day experimental period, the mice had free access to food and water on even days, while they underwent overnight fasting on odd days. Random blood glucose levels were measured on even days, while fasting blood glucose levels were assessed on odd days. Additionally, changes in the body weight of mice were monitored throughout the study period.

Before and after the treatment period, body composition analysis was performed using a Bruker minispec LF50 body composition analyzer to measure fat and lean mass proportions, which were normalized relative to body weight. On Day 22 of the experiment, whole blood (0.5 mL) was collected from each mouse into heparinized anticoagulant tubes or serum-separating tubes. After centrifugation (1000 g, 10 min, 4 °C), the serum and plasma were collected and stored for subsequent biochemical analyses.

Tissue collection was made following euthanasia, the heart, liver, spleen, lungs, kidneys, pancreas, and sciatic nerve were harvested, fixed in 10% neutral-buffered formalin, and processed for histological analysis. Serum levels of insulin, TG, TC, FFA, HDL-C, LDL-C, ALT, AST and γ-GT were quantified according to the manufacturer's instructions for the respective assay kits. For HbA_1c_ determination, 5 μL of whole blood was added to a sterile centrifuge tube and subsequently 60 μL of lysis buffer was added. The mixture was incubated at room temperature for 10 min to ensure complete lysis of the red blood cells. Following this, HbA_1c_ levels was determined using the colorimetric reaction of horseradish peroxidase according to the kit protocol.

Sections of the collected tissue specimens were obtained through a series of processes including dehydration, clearing, embedding, and sectioning. The heart, spleen, lungs, and kidneys were stained with H&E. The livers were stained with PAS, while the sciatic nerves were stained with LFB. The pancreas underwent immunohistochemical staining for insulin and glucagon. Using Image J, the area of insulin-positive signals was extracted; the insulin signal was quantified using intensity-weighted measurements and then normalized to the Placebo group. Similarly, Image J was used to extract glucagon-positive cells and calculate the proportion of alpha cells within the islets while normalizing these values to the Placebo group.

### Islet injury model

An islet injury model was established using multiple small-dose STZ (Beyotime) administrations, with placebo treatment serving as the control group. Specifically, a fresh 1 wt% STZ solution was prepared in a citrate/citrate buffer under light-protected conditions. After a 6-hour fasting period, C57 mice were intraperitoneally injected with STZ at a dose of 40 mg/kg. This procedure was repeated for five consecutive days, during which random blood glucose levels were monitored daily. Four weeks post-induction, the diabetic C57 mice were divided into three groups: the STZ control group (n = 8), which received placebo treatment after β-cell injury induction; the STZ + Suba@T-gel group, which received a single subcutaneous injection of Suba@T-gel (2.5 mg/mL, 0.2 mL, n = 8) and the STZ + Free Suba group, which received a single subcutaneous injection of Free Suba (2.5 mg/mL, 0.2 mL, n = 8). Next, blood glucose levels were continuously monitored for subsequent four weeks, after which an OGTT was conducted. Following this, the mice were euthanized and their pancreases were gathered. Finally, the pancreatic samples were sectioned and subjected to Ki67 and TUNEL immunofluorescent staining. The insulin-positive regions, Ki67-positive cells and total pancreatic areas were quantified using ImageJ software. The percentage of Ki67-positive cells was obtained by counting the ratio of Ki67-positive β cells to the total number of β cells. β-cell mass was determined by multiplying the ratio of insulin-positive area to total pancreatic area by the pancreatic weight.

### Statistical analysis

All data were expressed as mean ± standard deviation. Two-tailed Student's t-test was performed to analyze the statistical significance between two groups. We used “*” when *p* < 0.05, “**” when *p* < 0.01 and “***” when *p* < 0.001.

## Supplementary Material

Supplementary methods and figures.

## Figures and Tables

**Table 1:** Basic information of PLGA-PEG-PLGA triblock copolymers in this study.

Sample	*M_n_* (g/mol)^(a)^	LA/GA (mol/mol)^(a)^	*M_w_* (g/mol) ^(b)^	*Đ_m_* ^(b)^
Copolymer-I	1550-1500-1550	3.3: 1	4700	1.29
Copolymer-II	1840-1500-1840	3.2: 1	5300	1.22
Copolymer-III	2250-1500-2250	3.3: 1	6400	1.28
Copolymer-IV	1890-1500-1890	1.0: 1	5300	1.17
Copolymer-V	1890-1500-1890	9.5: 1	5000	1.20

^a^The *M*_n_ of PEG block was provided by Sigma-Aldrich. The *M*_n_ of PLGA block and the ratio of LA to GA were determined by ^1^H-NMR analysis. ^b^The ratio of weight-average MW (*M*_w_) to *M*_n_ is defined as *Đ*_m._ The *M*_w_s and *Đ*_m_ values of polymers were obtained by GPC.

**Table 2:** Pharmacokinetic parameters of different Suba formulations after a single injection.

	Free Suba (i.v.)	Free Suba (s.c.)	Suba@T-gel
*T_max_* (h)	1	24	24
*C_max_* (ng/mL)	24410	3546	1700
*t_1/2_* (h)	44.9	48.8	76.9
AUC_(0-last)_ (h·ng/mL)^a^	657241	186339	398068
MRT (h)^b^	23.8	22.4	209.4

^a^AUC_(0-last)_: area under the curve from time zero to the last sampling time point. ^b^MRT: mean retention time.

## Abbreviations

IDF: International Diabetes Federation
T2DM: Type 2 diabetes mellitus
GLP-1: Glucagon-like peptide-1
DPP-4: Dipeptidyl peptidase-4
GLP-1RA: Glucagon-like peptide-1 receptor agonist
HbA_1c_: Glycated hemoglobin
PEG: Poly(ethylene glycol)
PLGA: Poly(lactic acid-*co*-glycolic acid)
LA: lactic acid
GA: glycolic acid
GPC: Gel permeation chromatography
^1^H-NMR: Proton nuclear magnetic resonance
TEM: Transmission electron microscopy
CD: Circular dichroism
DLS: Dynamic light scattering
CMC: Critical micelle concentration
PVA: Poly(vinyl alcohol)
CCK-8: Cell Counting Kit-8
H&E: Hematoxylin and eosin
MRI: Magnetic resonance imaging
RB: Rhodamine B
AUC: Area under the curve
ICR: Institute of Cancer Research
OGTT: Oral glucose tolerance test
SD: Sprague-Dawley
STZ: Streptozotocin
TUNEL: Terminal deoxynucleotidyl transferase dUTP nick end labeling
TG: Triglyceride
TC: Total cholesterol
FFA: Free fatty acid
HDL-C: High-density lipoprotein cholesterol
LDL-C: Low-density lipoprotein cholesterol
PAS: Periodic acid-Schiff
LFB: Luxol Fast Blue
ALT: Alanine aminotransferase
AST: Aspartate aminotransferase
γ-GT: Gamma-glutamyl transferase
WT: Wild-type
FDA: Food and Drug Administration
PBS: Phosphate-buffered saline
ELISA: Enzyme-linked immunosorbent assay
