# Supplementary material for: Long-acting management of diabetes and associated complications using an injectable thermosensitive hydrogel incorporating IgG-conjugated GLP-1RA
Source: Theranostics. 2026 Jan 1;16(4):1833–54. doi: 10.7150/thno.120844 (PMC12680532; doi:10.7150/thno.120844)
Supplement: Supplementary file 1 — Supplementary methods and figures. [file thnov16p1833s1.pdf]

Supporting Information

Long-acting management of diabetes using an injectable thermosensitive hydrogel incorporating IgG-conjugated GLP-1RA

Hancheng Wang[1], Zhiyong Chen[1], Siyi Gu[1], Yaoben Wang[1], Yang Wang[1], Caiyun Gao[1], Jiayue Shi[1], Jiandong Ding[1], Qinghua Wang[2,3,*] & Lin Yu[1,*]

[1]State Key Laboratory of Molecular Engineering of Polymers, Department of Macromolecular Science, Fudan University, Shanghai 200438, China

[2]Department of Endocrinology and Metabolism, Huashan Hospital, Shanghai Medical School, Fudan University, Shanghai 200040, China

[3]Innogen Pharmaceutical Technology Co., Ltd., Shanghai 201203, China

*Corresponding authors

E-mail address: qh_wang@fudan.edu.cn (Q. Wang) and yu_lin@fudan.edu.cn (L. Yu)

Methods

Cytocompatibility measurement

For NIH/3T3 and βTC-6 cells (National collection of authenticated cell cultures, China), they were seeded in 96-well plates at a density of 4×10^3 cells per well. 3T3-L1 cells (National collection of authenticated cell cultures, China) were cultured in 6-well plates and induced for adipocyte differentiation. Once the cells adhered to the wall overnight, the culture medium was replaced with fresh medium containing Copolymer-II at varying concentrations (10-1000 μg/mL) and cultured for another 24 h (n = 6). After carefully aspirating the medium, fresh medium containing 10 % CCK-8 reagent (Beyotime) was added to each well according to the kit instructions, followed by an additional 4-hour incubation. Finally, cell viability was evaluated by measuring the absorbance of each well at 450 nm using a microplate reader. Cells cultured in medium without any additives were used as the negative control, while cells receiving the treatent of sodium dodecyl sulfonate (SDS) was used as the positive control.

Blood compatibility evaluation

1 mL of T-gel, Free Suba, or Suba@T-gel was added to the bottom of a 15-mL centrifuge tube and incubated in a 37 °C water bath for 30 min to allow gellation. Afterward, 10 mL of normal saline was added, and the samples were further incubated at 37 °C for 24 h with gentle agitation to facilitate extraction. Subsequently, the extract was filtered, and the clarified filtrate was collected for subsequent analysis. Next, 0.8 mL of the extract, along with a positive control (ultrapure water, UW) and a negative control (normal saline, NS), were placed into separate centrifuge tubes. Freshly collected rat blood, anticoagulated with EDTA, was added to each tube in a volume of 0.2 mL. The samples were then incubated in a shaking water bath at 37 °C for 1 h. After that, the tubes were centrifuged at 1500 rpm for 10 min, and the supernatant was collected. The absorbance of the supernatant was measured at 540 nm using a microplate reader. Hemolysis rates for the test materials were normalized by setting the absorbance corresponding to the positive control as 100% and the negative control as 0%.

Critical micelle concentration (CMC) determination

A series of polymer aqueous solutions with varying concentrations (ranging from 0.001 to 0.5 wt%) was prepared. Subsequently, 50 μL of 0.4 mM 1,6-diphenyl-1,3,5-hexatriene (DPH) methanol solution was added to each sample, ensuring a final DPH concentration of 4 μM in the system. After equilibrating the system at 25 °C for 12 h, the absorption spectra in the range of 320–420 nm were recorded using a UV-Vis spectrophotometer (TU-1950, Beijing Purkinje General Instrument Co. Ltd, China). Due to the sensitivity of the hydrophobic probe DPH to its microenvironment, when the probe enters the hydrophobic core of micelles formed at polymer concentrations above the CMC, a significant increase in absorbance is observed. The CMC value was obtained from the inflection point of the plot of the difference in absorbance at 377 nm and 400 nm versus the logarithmic concentration of the polymer.

Prediction of Suba amphiphilicity

By inputting the amino acid sequence of Suba into the website https://web.expasy.org/cgi-bin/protparam/protparam/, the hydrophilicity and hydrophobicity of the entire protein at different positions can be analyzed through the simulation of individual amino acid properties.

Synthesis of Cy7.5-modified Suba and RB-capped Copolymer-II

The Suba stock solution was lyophilized to obtain a powder, and 10 mg of the powder was redissolved in a 0.1 mol/L sodium bicarbonate ($NaHCO_3$) solution. Subsequently, 7.8 mg of Cy7.5 N-hydroxysuccinimide (Cy7.5-NHS) was completely dissolved in 0.5 mL of dimethyl sulfoxide. The fluorescent dye solution was slowly added dropwise to the Suba solution over a period of 1 h. The reaction was allowed to proceed for 12 h at room temperature under light-

protected conditions. Following the reaction, the aqueous mixture was transferred into a dialysis bag with a MW cutoff of 3000 Da. The dialysis was performed against deionized water, with the water being replaced every 8 h, for a total duration of 48 h. After dialysis, the solution was lyophilized to yield the final product.

For RB-modified Copolymer-II, 5 g of Copolymer-II were dissolved in toluene, and the residual water was removed by azeotropic distillation. In a round-bottom flask, two equivalents of RB and three equivalents of 1-ethyl-3-(3-dimethylaminopropyl)carbodiimide along with 4-dimethylaminopyridine were added. The mixture was thoroughly dissolved in 20 mL of anhydrous dichloromethane and reacted under an inert atmosphere for 36 h. After the reaction, the mixture was washed three times by 0.1 mol/L $NaHCO_3$ solution, and the organic phase was extracted three times with anhydrous dichloromethane. The collected organic phase was dried over anhydrous sodium sulfate, and the solution was concentrated using a rotary evaporator. The concentrated solution was precipitated with anhydrous ether, and the precipitate was collected and lyophilized to yield the final product.

Metabolism study

During a 12-hour nighttime cycle, db/db mice were housed in metabolic cages, and the excreted waste during this period was collected and weighed.

Supplementary Figures and Tables

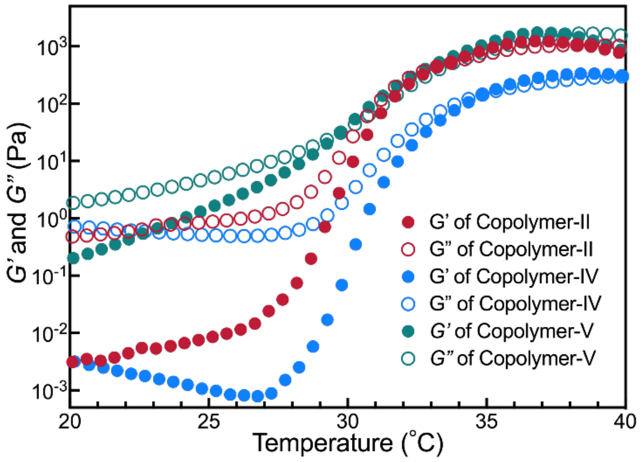

Figure S1. Rheological curves of aqueous soluitons of Copolymer-II, IV and V as a function of temperature.

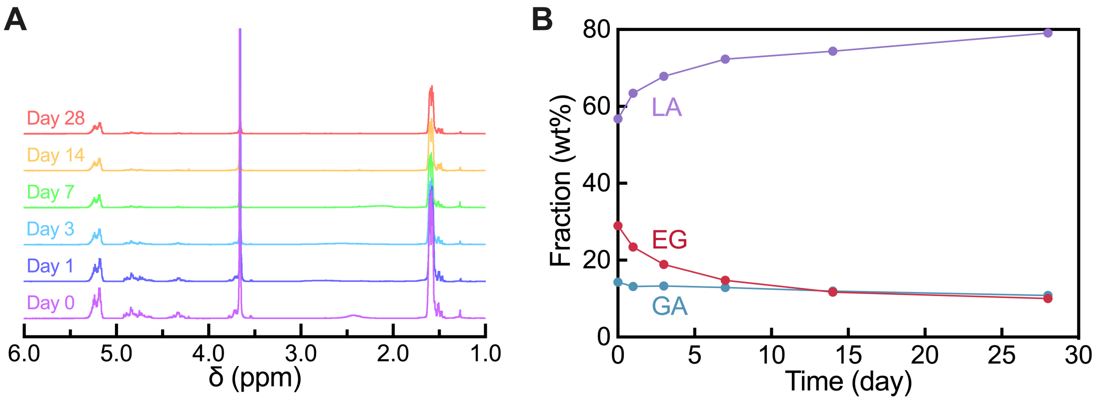

Figure S2. A) ^{1}H NMR spectra of residual T-gel collected at different time points and subsequently freeze-dried. B) Changes in the mass fractions of different polymeric units over time.

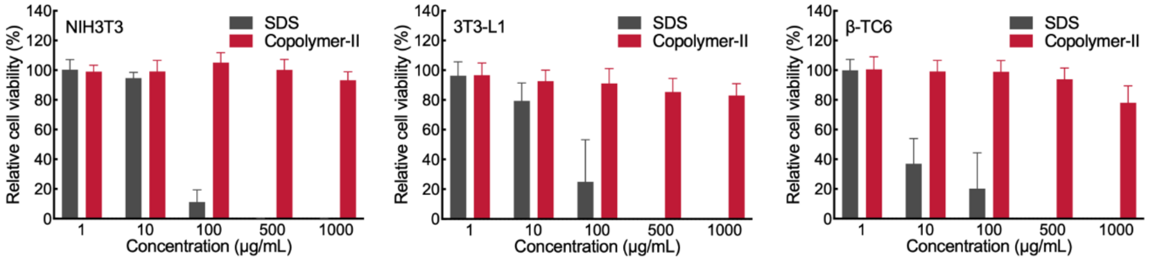

Figure S3. *In vitro* cytotoxicity evaluation of Copolymer-II. Data are represented as mean ± standard deviation (n = 8).

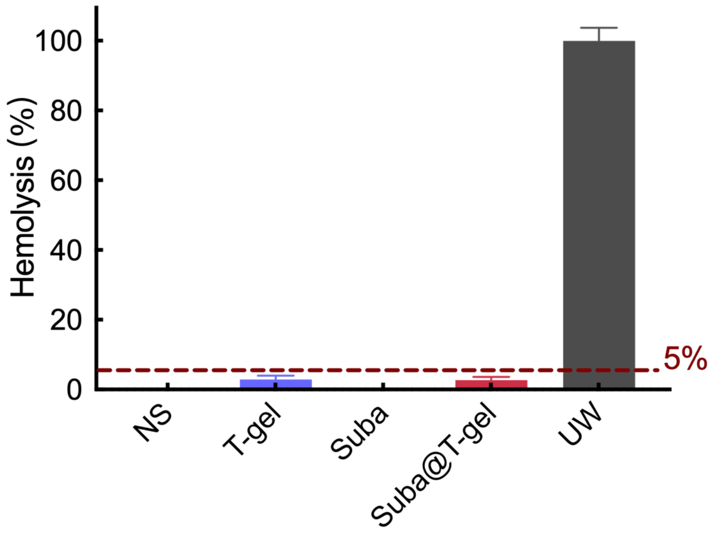

Figure S4. Blood compatibility of extracts of T-gel, Free Suba, and Suba@T-gel. NS and UW were used as the negative and positive controls, respectively (n = 3).

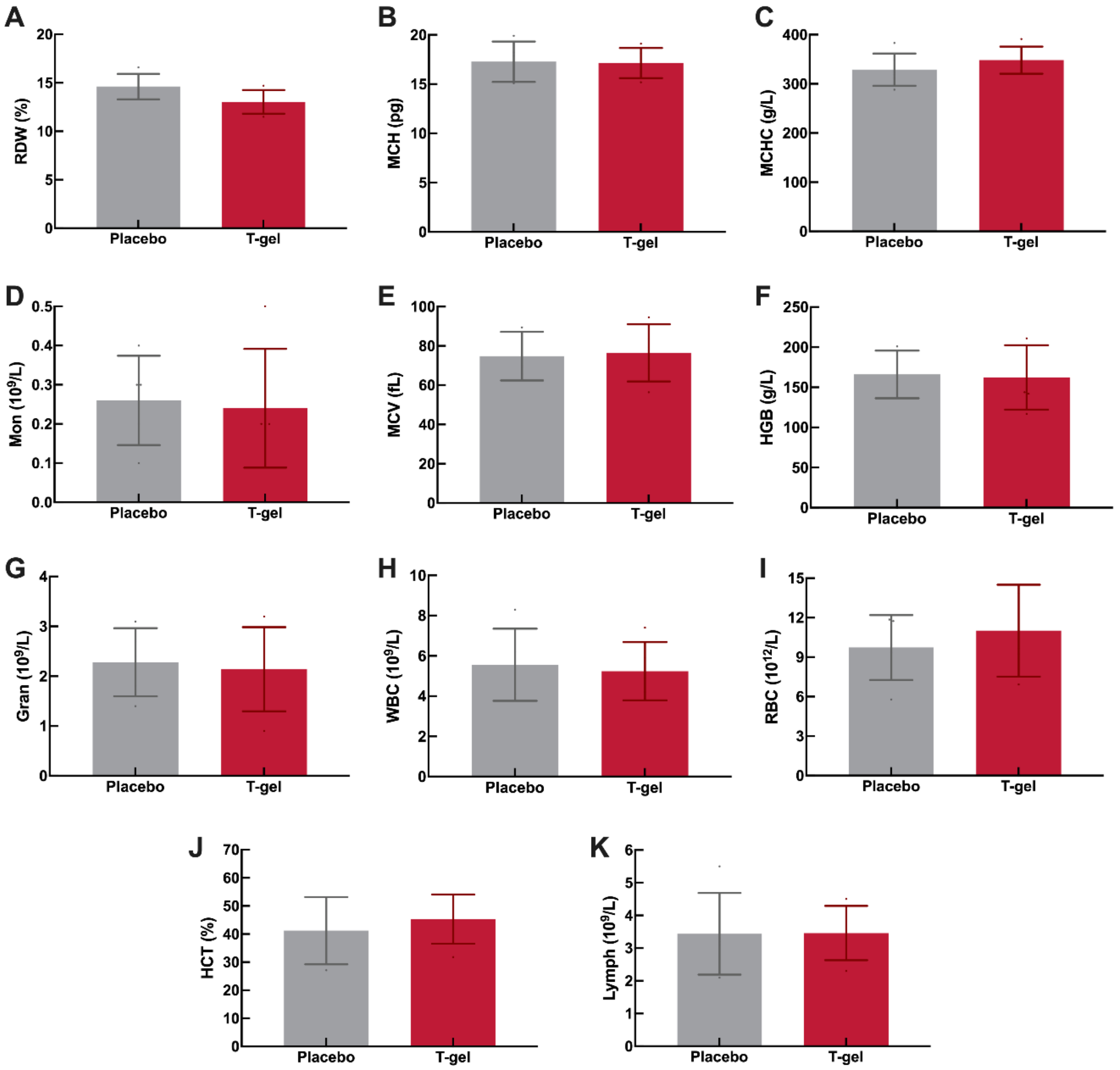

Figure S5. Blood routine examination of mice on day 28 after subcutaneous injection of T-gel. Data are represented as mean ± standard deviation (n = 6). A) RDW: Red Cell Distribution Width, B) MCH: Mean Corpuscular Hemoglobin, C) MCHC: Mean Corpuscular Hemoglobin Concentration, D) Mon: Monocytes, E) MCV: Mean Corpuscular Volume, F) HGB: Hemoglobin, G) Gran: Granulocytes, H) WBC: White Blood Cells, I) RBC: Red Blood Cells, J) HCT: Hematocrit, K) Lymph: Lymphocytes.

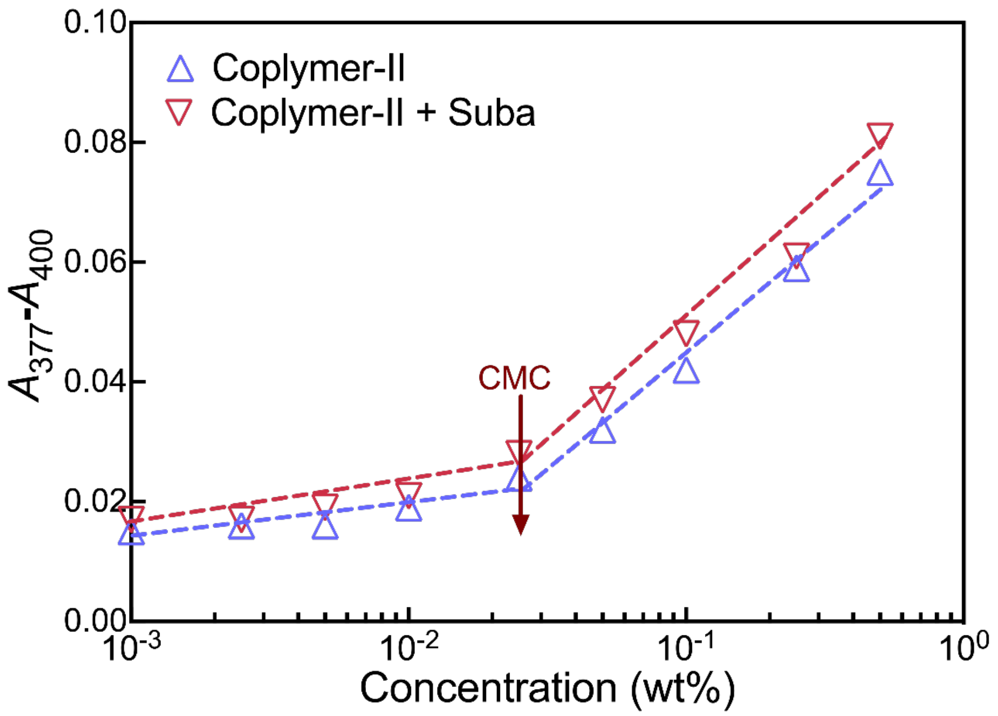

Figure S6. CMCs of Copolymer-II in water with or without Suba.

Table S1. Specific gravity analysis of secondary structure of Suba and BSA.

	Suba	Suba + PEG	Suba + PLGA-PEG-PLGA	BSA	BSA + PEG	BSA + PLGA-PEG-PLGA
α-Helix	90.1%	88.5%	20.2%	99.3%	99.3%	99.3%
β-Turn	8.4%	8.2%	19.7%	1.8%	1.8%	1.8%
Random coil	6.0%	6.5%	41.9%	2.2%	2.2%	2.2%
Parallel	1.0%	1.7%	12.1%	0	0	0
Anti-parallel	1.2%	1.3%	29.3%	0.5%	0.5%	0.5%

Note: The data were calculated using software included with the CD instrument.

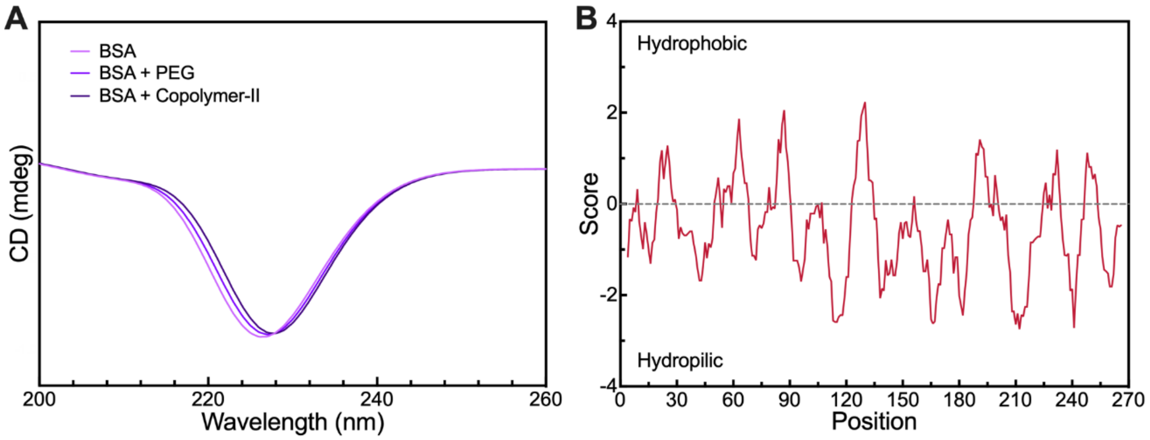

Figure S7. Secondary structure properties of different proteins. A) CD spectra of BSA with or without PEG/Copolymer-II. B) Prediction of hydrophilicity and hydrophobicity for each amino acid residue of Suba. The prediction was performed using the website https://web.expasy.org/cgi-bin/protparam/protparam.

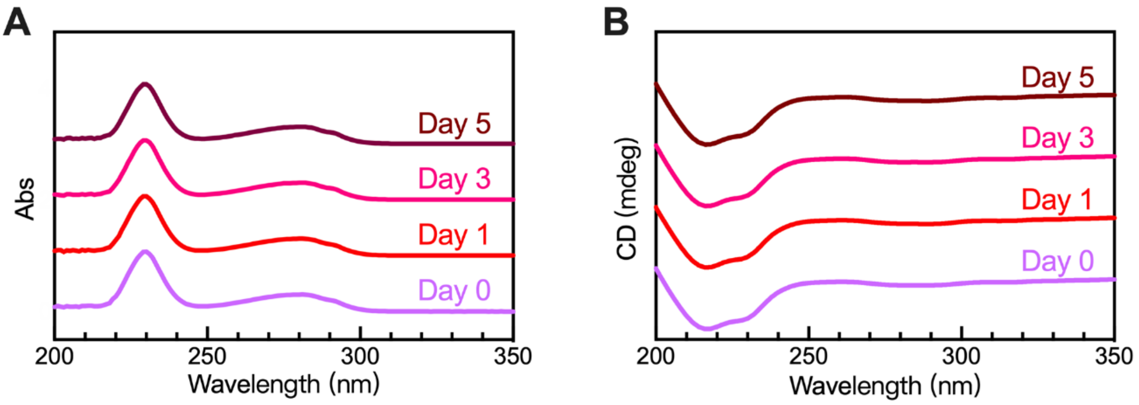

Figure S8. Stability evaluation of Suba. A) Ultraviolet-visible spectra and B) CD spectra of Suba solution incubated in a water bath at 37 °C for different time points.

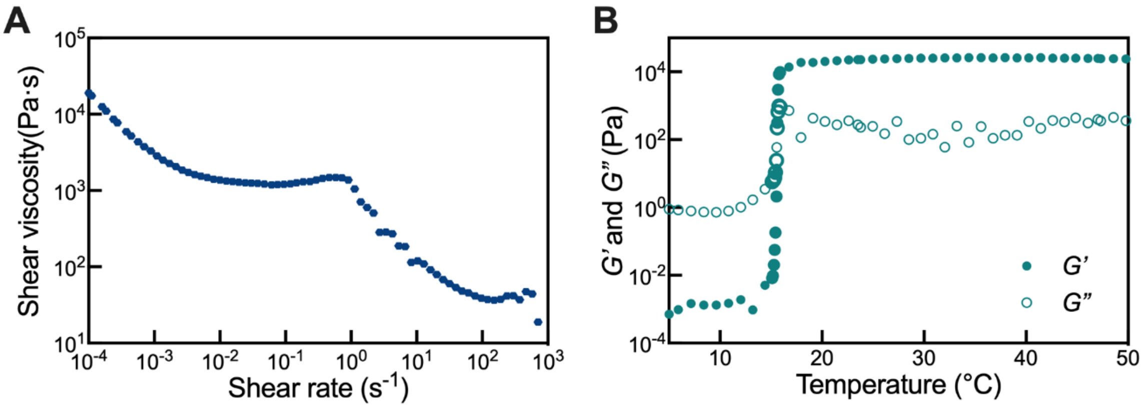

Figure S9. Rheological properties of different hydrogels. A) Changes in shear viscosity of PVA-gel (10 wt%) with shear rate, indicating its shear-thinning properties. PVA-gel was obtained by a freeze-thaw method. B) Reological curves of commercial F127-gel (20 wt%) as a function of temperature.

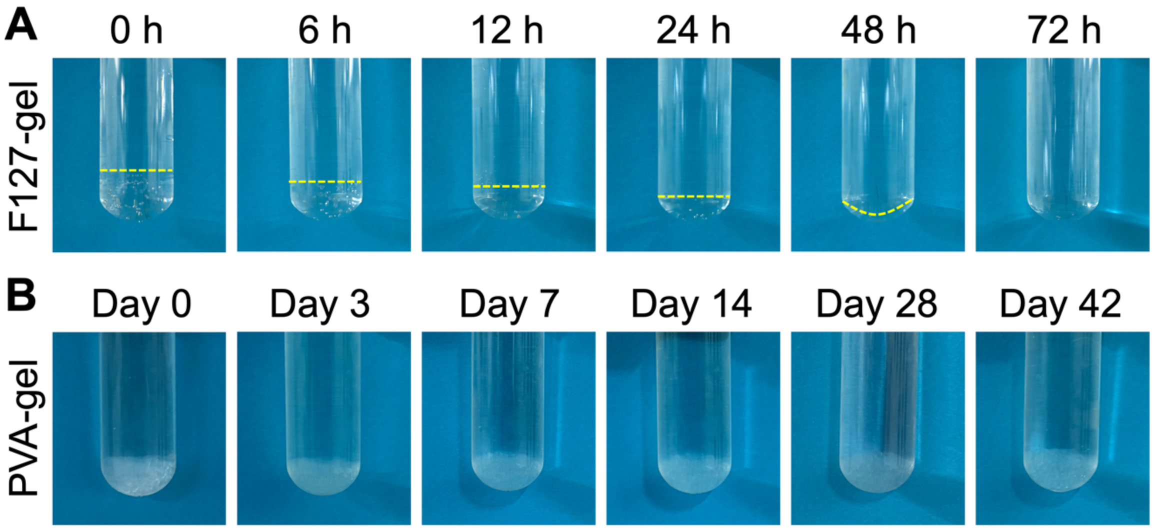

Figure S10. Optical images of A) F127-gel and B) PVA-gel after incubation in the release medium for various periods in a shaking water bath at 37 °C. The yellow dotted lines represent the boundary of F127-gel.

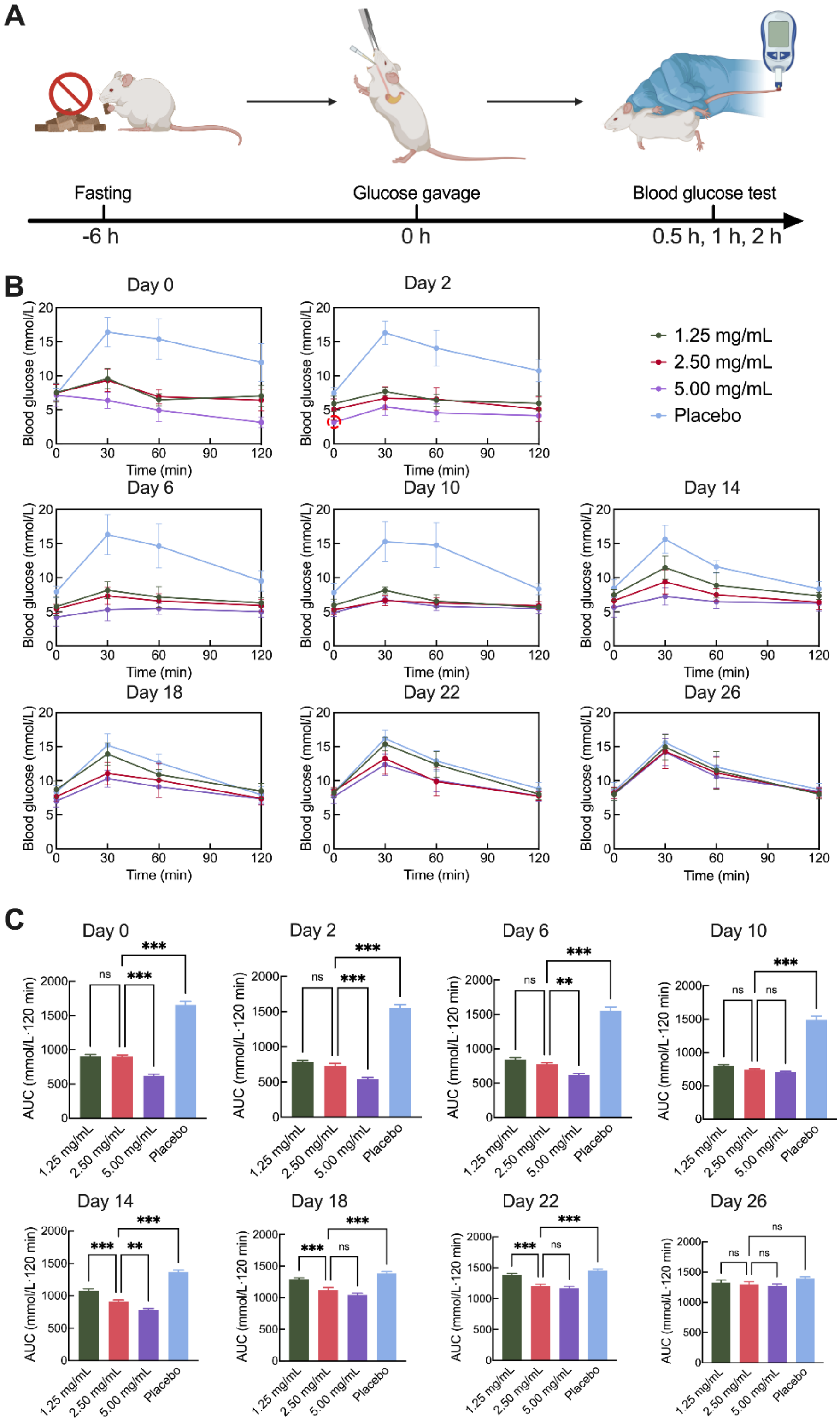

Figure S11. OGTTs in normal ICR mice. A) Schematic diagram of OGTT. B) Blood glucose curves of mice treated with Suba@T-gel formulations containing different concentrations of Suba at different time points post-glucose administration and C) statistical analysis of corresponding AUC (n = 6).

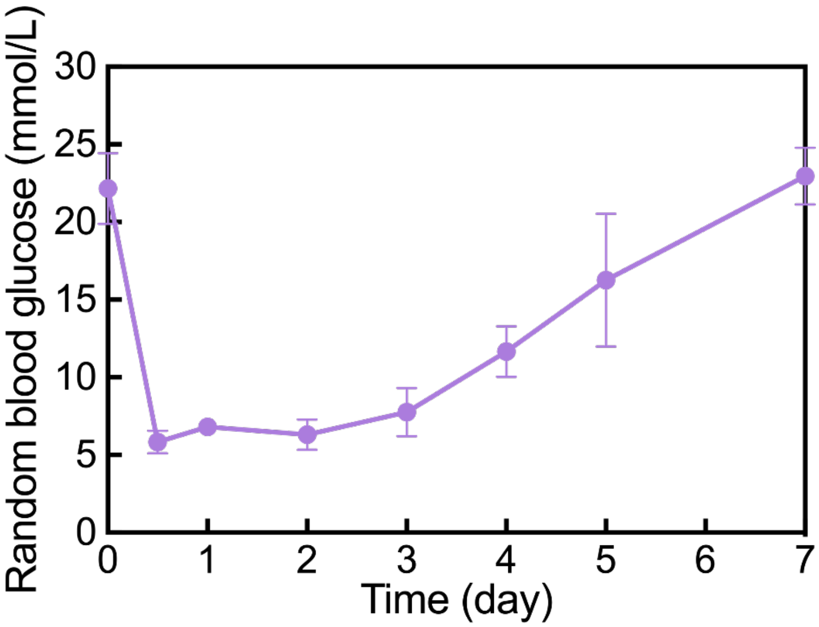

Figure S12. Random blood glucose levels of db/db mice after a single injection of Free Suba.

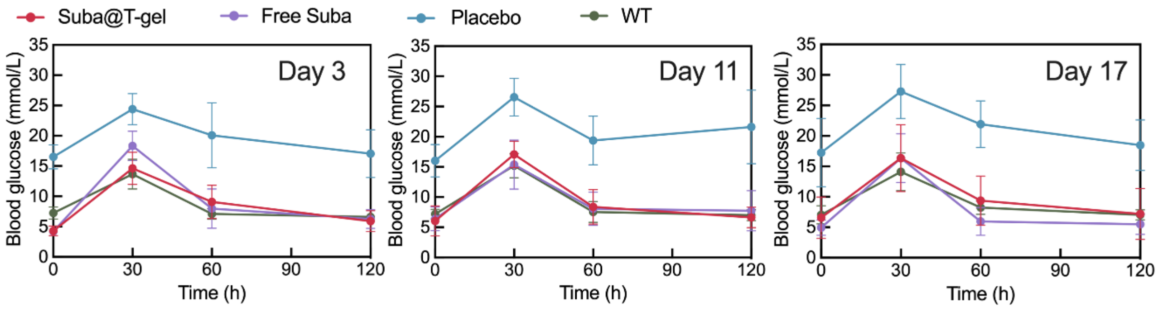

Figure S13. OGTTs of db/db mice conducted on day 3, 11, and 17 (n = 8).

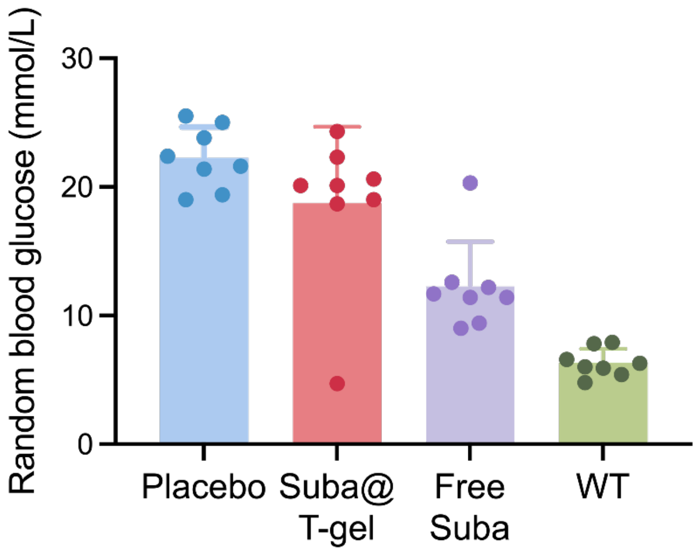

Figure S14. Random blood glucose levels of mice on day 22 (n = 8).

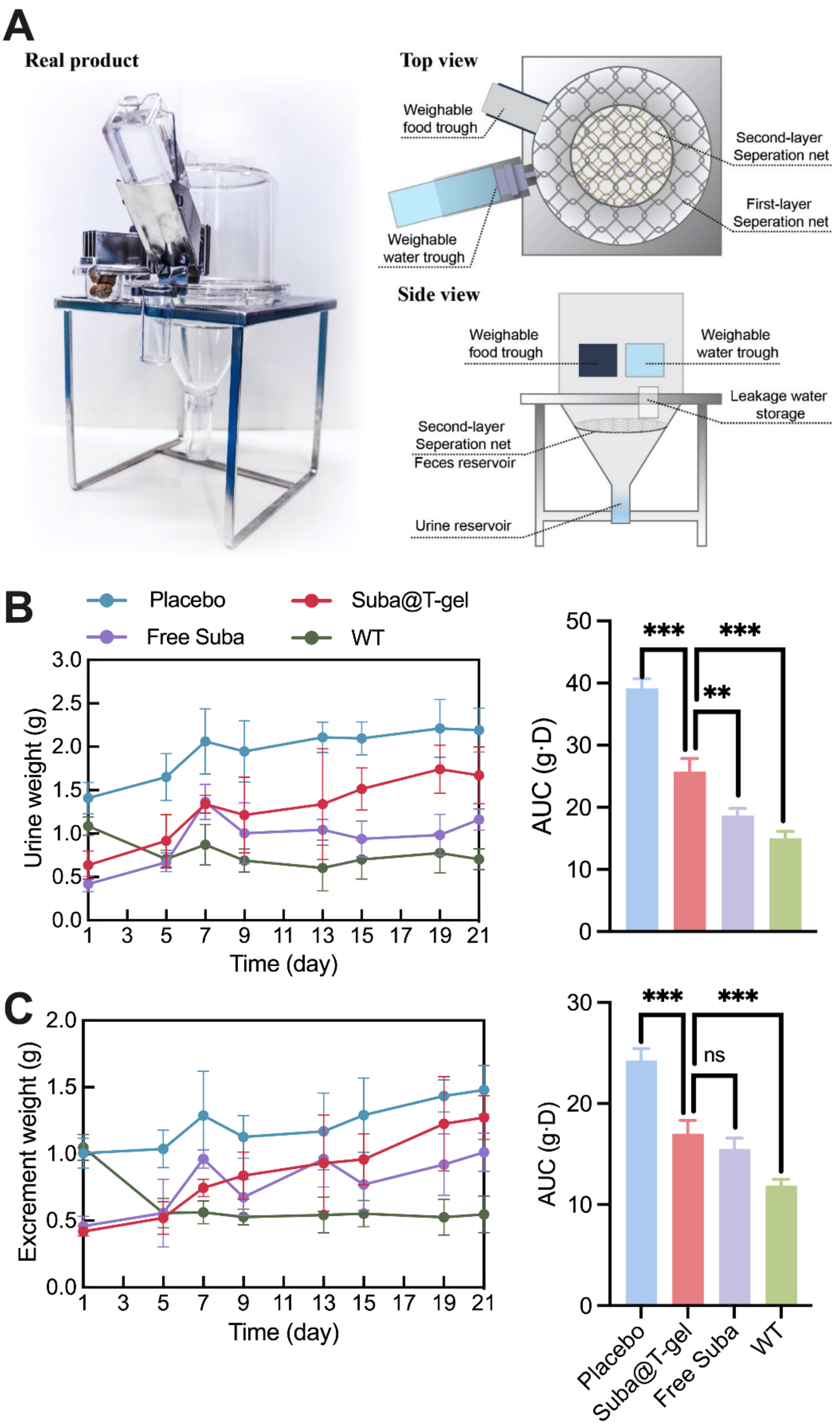

Figure S15. Metabolic profile of mice during treatment assessed using metabolic cages. A) Schematic diagram of metabolic cage. B-C) Metabolism of db/db mice after various treatments and statistical analysis of corresponding AUC (n = 4).

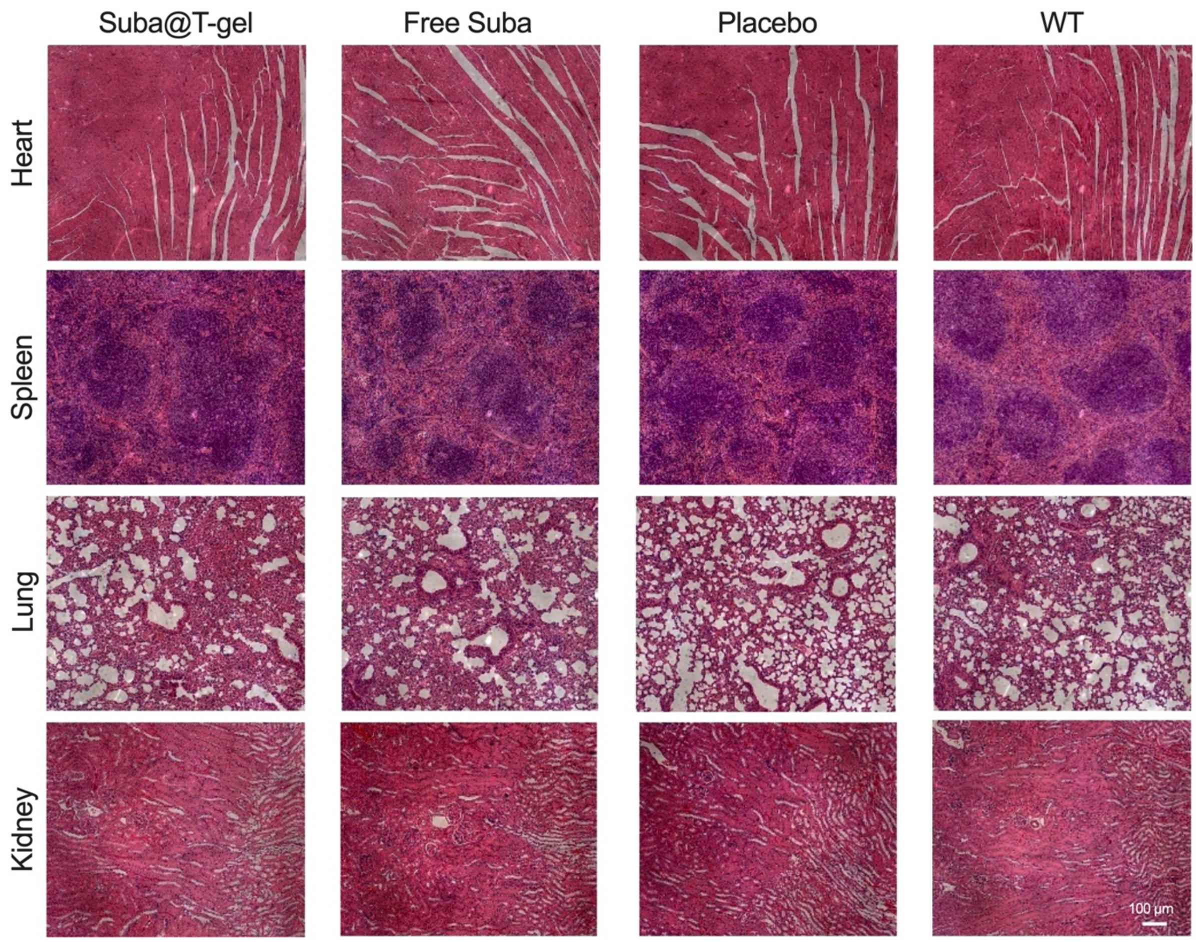

Figure S16. H&E-stained slices of major organs collectd from db/db mice at the end of various treatments.
